# Relationships between Motor Proficiency and Academic Performance in Mathematics and Reading in School-Aged Children and Adolescents: A Systematic Review

**DOI:** 10.3390/ijerph15081603

**Published:** 2018-07-28

**Authors:** Kirstin Macdonald, Nikki Milne, Robin Orr, Rodney Pope

**Affiliations:** 1 Physiotherapy Program, Faculty of Health Sciences and Medicine, Bond University, Gold Coast, QLD 4229, Australia; nmilne@bond.edu.au (N.M.); rorr@bond.edu.au (R.O.); rpope@csu.edu.au (R.P.); 2 School of Community Health, Charles Sturt University, Thurgoona, NSW 2640, Australia

**Keywords:** physical activity, motor proficiency, academic performance, mathematics, reading, children, adolescents

## Abstract

Positive associations exist between physical activity, cognition, and academic performance in children and adolescents. Further research is required to examine which factors underpin the relationships between physical activity and academic performance. This systematic review aimed to identify, critically appraise, and synthesize findings of studies examining relationships between motor proficiency and academic performance in mathematics and reading in typically developing school-aged children and adolescents. A systematic search of electronic databases was performed to identify relevant studies. Fifty-five eligible articles were critically appraised and key data was extracted and synthesized. Findings support associations between several components of motor proficiency and academic performance in mathematics and reading. There was evidence that fine motor proficiency was significantly and positively associated with academic performance in mathematics and reading, particularly during the early years of school. Significant positive associations were also evident between academic performance and components of gross motor proficiency, specifically speed and agility, upper-limb coordination, and total gross motor scores. Preliminary evidence from a small number of experimental studies suggests motor skill interventions in primary school settings may have a positive impact on academic performance in mathematics and/or reading. Future research should include more robust study designs to explore more extensively the impact of motor skill interventions on academic performance.

## 1. Introduction

Early childhood curriculum and pedagogical approaches aim to promote holistic attitudes to teaching and learning which recognise the important contribution a child’s physical, cognitive, social, and emotional development has on their learning and readiness to start school [1]. However, upon school entry, the primary focus of learning often shifts to developing students’ academic skills, particularly in numeracy and literacy [2]. Consequently, there may be less dedicated time in the school curriculum for encouraging the ongoing physical development of students, which ideally aims to support the acquisition of motor skills and foster positive attitudes towards physical activity (PA) [3]. The disparity between the competing priorities of physical versus academic development has been debated in recent years due to increasing awareness of the global public health implications of growing physical inactivity and sedentary time in youth [4].

The importance of PA is widely recognized, with regular and adequate levels of PA leading to improvements in muscular and cardiorespiratory fitness (CRF) and bone health, along with a reduction in levels of cardiovascular disease, cancer, and diabetes [4,5]. Furthermore, social, emotional, and intellectual benefits of PA, such as improved self-esteem and confidence and enhanced concentration, have also been reported [4,5]. However, recent surveys suggest that only approximately 19% of Australian children and young people aged five to 17 years meet the recommended 60 min of moderate to vigorous PA per day [6]. Several reviews [7,8,9,10,11,12,13] have consistently reported significant, positive associations between PA and cognition and/or academic performance in children and adolescents. However, it remains unclear as to the exact type, frequency, duration, and intensity of PA that is required to impact cognitive functioning in children and adolescents [8,14].

The majority of studies investigating the impact of PA on cognition and academic performance in school-aged children have measured either overall levels of PA (objectively, using accelerometers [15], or subjectively, through questionnaires [16]) or health-related physical fitness, in particular CRF [8]. For example, a recently published systematic review by Donnelly et al. [8] comprehensively summarized the findings of studies examining the relationships between PA, fitness, cognitive function, and academic achievement. The synthesis included a summary of findings from 27 observational studies that examined the relationship between physical fitness and academic achievement in children aged five to 13 years, demonstrating largely positive findings, although it highlighted several limitations in relation to study quality and reporting that resulted in inconsistent findings. Notably, the components of physical fitness measured by these studies included CRF, muscular strength and endurance, flexibility, and body composition. However, as engagement in PA is dependent not only on health-related physical fitness but also on performance-related physical fitness, which we have termed ‘motor proficiency’ in this review, further examination of the relationships between motor proficiency, cognition, and academic performance is warranted, as reviews to date on these relationships have been scant [17].

Over several decades, researchers have reviewed the impacts of perceptual motor programs on the academic performance of school students, providing inconsistent findings and insufficient evidence, as many of the studies had notable methodological flaws [18,19]. Perceptual motor skills require the integration of sensory input (visual, auditory, and kinaesthetic) with fine or gross motor responses [19]. More recently, the focus has shifted to investigating the relationship between fine motor skills and academic performance, given emerging findings that fine motor skills may be a significant indicator for school readiness [20,21]. A systematic review published in 2015 by van der Fels et al. [22] summarized the findings from studies examining the relationship between motor and cognitive skills in typically developing school children and also noted inconsistent findings, with either no association reported in the literature or insufficient evidence for or against many associations between motor and cognitive variables. However, the authors [22] highlighted that weak to strong evidence was found to support the relationship between more complex motor skills, such as bilateral body coordination and cognitive skills.

In recent years, several systematic reviews [8,13,23,24] have evaluated the impact of school-based PA interventions on the educational and health outcomes of school students. For example, one approach of providing PA opportunities to students during the school day, distinct from physical education (PE) classes and break times, is classroom-based PA [24,25]. Classroom-based PA may involve the integration of PA into academic lessons or may include incorporating PA into the regular classroom routine without an academic focus [24,25]. However, research evaluating the impact of classroom-based PA on learning is still in its infancy, with inconsistent findings from a small number of studies with varying methodological quality and study designs reported in recently published systematic reviews [23,24]. However, there is preliminary evidence to suggest that classroom-based PA, particularly with an academic focus, may have a positive impact on both academic performance and overall levels of PA [8,13,23,24]. To date, no systematic review on this topic has specifically evaluated the impact of motor proficiency-related interventions in the school setting on academic performance and motor proficiency outcomes.

The present systematic review builds on the reviews published by van der Fels et al. [22] and Donnelly et al. [8] by examining, in greater detail than previously reported, the underlying domains of motor proficiency (i.e., fine and gross motor skills) that may be associated with academic performance of school students. This review also expands on findings from reviews investigating the impact of classroom-based PA on learning outcomes by specifying motor skills as the type of PA being evaluated and academic performance in mathematics and reading as the learning outcomes of interest. Academic performance in mathematics and reading has specifically been chosen for this review due to the priority for students to develop foundational skills in numeracy and literacy upon entry to school. Therefore, the overall objective of this systematic review was to identify, critically appraise, and synthesize the findings of studies examining the relationship between motor proficiency and academic performance in mathematics and reading in typically developing school-aged children and adolescents. Two main aims were developed to address this objective: (1) to determine whether a relationship exists between motor proficiency and academic performance in mathematics and reading in typically developing school-aged children and adolescents; and (2) to determine whether motor proficiency-related interventions impact academic performance in mathematics and reading in typically developing school-aged children and adolescents.

## 2. Materials and Methods

### 2.1. Identification of Studies

To identify relevant studies, a comprehensive search of health and education databases was undertaken using a four-step approach, and documented in accordance with the Preferred Reporting Items for Systematic Reviews and Meta-Analyses (PRISMA) protocol [26]. Firstly, electronic databases (EBSCO, PubMED, PsychINFO, and Web of Science) were searched on 21 February 2018 using key search terms to identify literature relevant to this topic. The key search terms were: ((motor AND (proficiency OR competency OR skill* OR development OR ability OR performance OR movement OR gross OR fine)) AND (“academic performance” OR “academic achievement” OR “academic grades” OR math* OR numeracy OR reading OR literacy) AND (child* OR adolescen* OR “school student”)). The search terms for each database are available upon request from the authors.

### 2.2. Screening and Selection

Following removal of duplicates, the titles and abstracts of the remaining studies identified in the database searches were screened with reference to pre-determined inclusion and exclusion criteria to assess their potential eligibility for inclusion in this review. Studies in which the abstract clearly indicated the study would be ineligible for inclusion in the review were eliminated, but studies for which any doubt regarding eligibility existed and those considered likely to be eligible were retained; full text copies of these studies were subsequently obtained. The full text copies of these remaining studies were then independently assessed for eligibility for inclusion in the review by one of the reviewers (K.M.), using the pre-determined inclusion and exclusion criteria described below. For inclusion in this review, studies were required to meet the following criteria:

(1) The study population had to include typically developing school children aged between four and 18 years.
(2) For observational studies, at least one component of motor proficiency had to have been objectively measured and reported. Motor proficiency, as described by Bruininks [27], incorporates the following components: fine motor precision, fine motor integration (visual motor integration), manual dexterity, upper limb coordination, bilateral coordination, balance, speed and agility, and strength. For experimental studies, the intervention had to specifically incorporate a component of motor proficiency delivered during the school day (e.g., academic and/or PE lessons), designed to impact academic performance in mathematics and/or reading.
(3) For observational studies, associations between an objective measure of fine or gross motor proficiency AND an objective measure of academic performance (specifically in mathematics, reading or their underlying constructs) had to have been reported. Appropriate statistical analyses for reporting associations included correlations and regression or structural equation modelling. For experimental studies, the pre-test and post-test values of motor proficiency and academic outcomes in mathematics and reading for both control and experimental groups or a measure of treatment effect on academic performance needed to be reported.
(4) Studies had to be either observational or experimental in design.
(5) Studies had to have been published after January 2000, due to the methodological limitations in studies published prior to 2000 previously described [18,19].

Following application of these inclusion criteria, five exclusion criteria were applied in the study selection process, these being:

(1) Studies involving a population of school-aged children diagnosed with either an intellectual disability or a neurodevelopmental disorder (e.g., specific learning disorder, developmental coordination disorder, attention deficit hyperactivity disorder or autism spectrum disorders), as defined by the Diagnostic and Statistical Manual of Mental Disorders (5th Edition) [28].
(2) For experimental studies, intervention(s) focused on health-related fitness (e.g., CRF) or general physical activity rather than an intervention specifically focused on motor proficiency *or* motor proficiency-related interventions that were implemented outside school hours.
(3) Studies reporting motor outcomes that included only an overall fitness score (i.e., a combination of performance and health-related physical fitness).
(4) Studies reporting academic outcomes in terms of an overall academic performance score only (i.e., a combination of mathematics and reading) but not an individual score for mathematics and/or reading.
(5) Studies published in a language other than English, where a translated version could not be sourced.

Eligible studies were retained and included in the review and were subject to subsequent quality assessment, data extraction, and synthesis. Reference lists of all eligible articles were also reviewed, and potentially eligible studies not previously identified were sourced in full text and subjected to the same selection process.

### 2.3. Critical Appraisal of Methodological Quality

Two authors (K.M., N.M.) independently assessed the methodological quality of included studies using the Downs and Black checklist [29]. The checklist includes five subcategories including: reporting quality, external validity, internal validity (bias and confounding), and statistical power. The checklist consists of 27 items, of which 25 items use a scoring system of 1 = Yes, 0 = No, or 0 = Unable to determine. Two of the questions have a greater scoring range. Item 5 is normally scored from 0 to 2 points, where 1 point is awarded for partially detailed confounding factors and 2 points are awarded for comprehensively detailed confounding factors. Item 27 is normally scored from 0 to 5 points based on the statistical power of the study. For this review, item 27 was modified to be scored with either 1 point if the study outlined the statistical power or basis for the sample size for the study, or 0 points if the study did not describe the statistical power or basis for the sample size. This modified approach has been previously reported [30]. To provide a rating of quality, the rating system described by Kennelly [31] was used, and was slightly adapted as discussed below. Kennelly’s rating system is based on the original scoring system reported by Downs and Black [29], however, due to the modifications made to the checklist in the present review, critical appraisal scores of studies were first converted to percentages before these percentages were used to assign a quality rating to each of the studies.

To address the first aim of the current review, which was to determine whether a relationship exists between motor proficiency and academic performance in mathematics and reading in typically developing school-aged children, the methodological quality of included observational studies was assessed. However, items 4, 8, 13, 14, 15, 19, 23, and 24 of the Downs and Black checklist [29] were not assessed for these studies, as these items were specifically relevant to the methodologies used in experimental studies, and not to methods used in observational study designs. For example, item 4 asks: ‘Are the interventions of interest clearly described?’ [29]. To provide a rating of quality, the raw total score from the modified Downs and Black checklist for each observational study was converted to a critical appraisal percentage (CAP) by dividing the raw score by 20 points (the maximum possible score) and multiplying it by 100. This method for modifying the Downs and Black checklist [29] for observational studies was previously published by Lyons et al. [32]. Studies were then allocated a methodological quality rating, with studies achieving a CAP ≥71% classified as ‘good’ quality, 54–70% classified as ‘fair’ quality, and ≤53% classified as ‘poor’ quality [31].

To address the second aim of the review, to determine whether motor proficiency-related interventions impact academic performance in mathematics and reading in typically developing school-aged children and adolescents, the methodological quality of eligible experimental studies was assessed using all items of the Downs and Black checklist [29]. To provide a rating of quality, the raw score for each experimental study was converted to a critical appraisal percentage (CAP) by dividing each study’s raw score by 28 points, and multiplying it by 100. All studies were then allocated a methodological quality rating score, with studies achieving a CAP ≥71% classified as ‘good’ quality, 54–70% classified as ‘fair’ quality and ≤53% classified as ‘poor’ quality [31]. This method for determining the quality rating score has been previously described by Terry et al. [33].

To determine the level of agreement between the critical appraisal scores derived by the two independent reviewers (K.M. and N.M.) when applying the modified Downs and Black checklist, a kappa coefficient was calculated by a third author (R.O.) and graded using methods previously reported by Viera and Garrett [34]. Following this process, any discrepancies in critical appraisal scores between the two authors (K.M. and N.M.) which could not be resolved by discussion and consensus were moderated by the third author (R.O.).

### 2.4. Data Extraction

Key data from each of the eligible studies was extracted by a single reviewer (K.M.) into a Microsoft Excel spreadsheet constructed to standardize the data extraction. Another reviewer (R.P.) checked the extracted data. The data elements included: (1) author and study design; (2) characteristics of the study sample; (3) the objective outcome measures used to assess fine or gross motor proficiency; (4) the objective outcome measures used to assess academic performance in mathematics and reading; (5) any covariates (confounding factors) included in analyses; (6) characteristics of the intervention (where relevant); and (7) summary of main findings from each study. The data elements that were extracted from observational studies are depicted in the headings of Supplementary Table S1 and the data elements extracted from experimental studies are depicted in the headings of Supplementary Table S2.

### 2.5. Synthesis

The first aim of the review was to examine the relationships between motor proficiency and academic performance in mathematics and reading, and thus associations between these variables in each included study (when reported) were extracted and considered in a critical narrative synthesis of key findings from the included studies. For the purposes of the synthesis, motor proficiency, as described by Bruininks [27], was categorized into the eight motor subtests of the Bruininks-Oseretsky Test of Motor Proficiency (2nd Edition) (BOT-2). The BOT-2 is a valid and reliable standardized assessment tool, suitable for evaluating the fine and gross motor proficiency of individuals aged four to 21 years [27]. Key findings extracted from included studies for the following components of fine motor proficiency were considered in the synthesis: (1) fine motor precision; (2) fine motor integration; (3) manual dexterity; and (4) total fine motor score. Key findings extracted for the following components of gross motor proficiency were also considered in the synthesis: (1) upper limb coordination; (2) balance; (3) bilateral coordination; (4) speed and agility; (5) strength; and (6) total gross motor score. Findings for total motor proficiency score, representing the sum of fine and gross motor scores, were also considered in the synthesis.

During the synthesis of key findings, interpretation of the strengths of the correlations (r) between the different areas of motor proficiency and academic performance in mathematics and reading was guided by a rating scale described by Evans [35] as follows: r = 0.00–0.19 (very weak), r = 0.20–0.39 (weak), r = 0.40–0.59 (moderate), r = 0.60–0.79 (strong), and r = 0.80–1.0 (very strong). Significant and non-significant associations reported in studies included in the review were then summarized and coded using an approach adapted from that originally described by Sallis et al. [36] for observational studies investigating relationships between variables in the field of public health. For each component of motor proficiency, a percentage was calculated to represent the proportion of reported associations between that component of motor proficiency and academic performance that reached statistical significance. The overall result was then classified as: (1) no association, coded ‘0’ (indicating 0–33% of reported associations reached statistical significance); (2) inconsistent/uncertain association, coded ‘?’ (indicating 34–59% of reported associations reached statistical significance or less than four studies examined the relationship); or (3) a positive or negative association coded ‘+’ or ‘−’ (indicating that ≥60% of reported associations reached statistical significance, based on results of four or more studies). In the latter case, when four or more studies with a Kennelly rating [31] of ‘fair’ or ‘good’ methodological quality reported a statistically-significant positive or negative association between a given motor proficiency variable and a particular academic performance variable, the positive or negative association was coded ‘+ +’ or ‘− −‘, respectively, representing the fact that strong evidence, based on multiple studies of ‘fair’ or ‘good’ methodological quality, supported the significant association. This latter approach is similar to that used by Lubans et al. [37] and Cliff et al. [38] in their systematic reviews.

To address the second aim of the review, a critical narrative synthesis was also undertaken to synthesize the key findings from the included experimental studies that investigated whether motor proficiency-related interventions impacted academic performance in typically developing school-aged children and adolescents. Given the heterogeneity between studies in their design, outcome measures and study quality, a meta-analysis was not conducted.

## 3. Results

### 3.1. Included Studies and Study Characteristics

**Figure 1:**
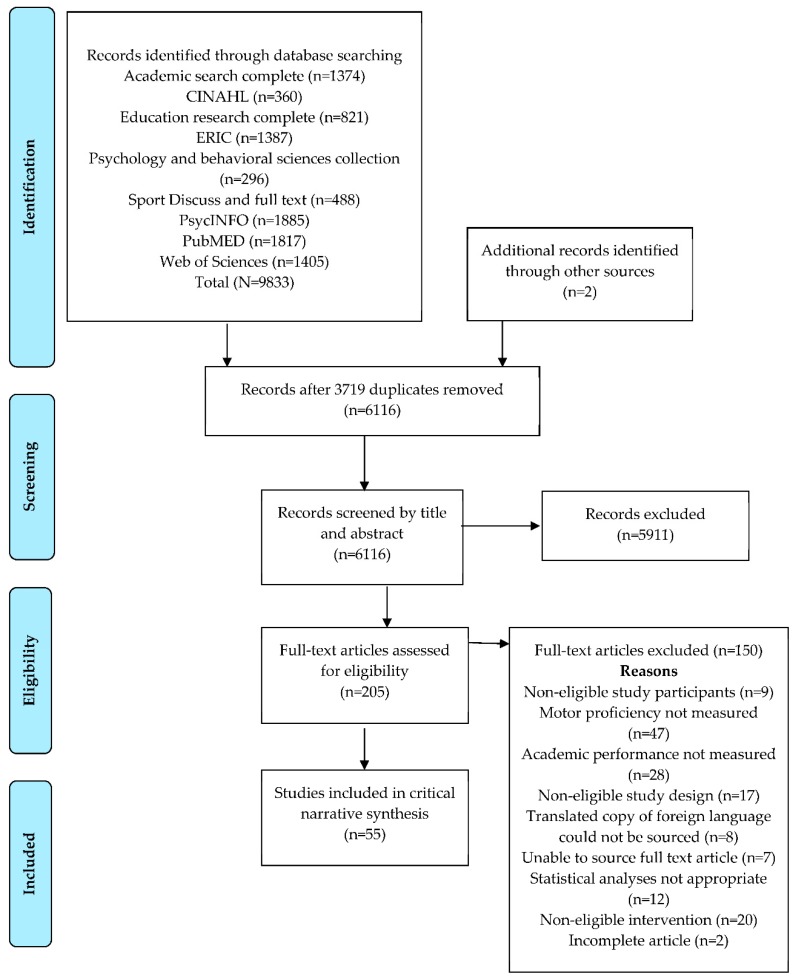
Preferred Reporting Items for Systematic Reviews and Meta-Analyses (PRISMA) flow diagram [26] depicting results of the search, screening, and selection processes.

The PRISMA diagram [26] in Figure 1 summarizes the results of the four-step approach taken to identify, screen, and select studies for inclusion in this review. Following completion of screening and selection, a total of 55 studies were deemed eligible for inclusion. Table S1 summarizes the key characteristics extracted from the 51 observational studies (26 longitudinal and 25 cross-sectional) included in the review. Table S2 summarizes the key characteristics extracted from the four experimental studies (one cluster randomized controlled trial and three quasi-experimental studies).

As noted in Tables S1 and S2, just over half (56%) of the studies included in the review had been published since 2014. Total participant sample sizes in studies included in the review varied between 36 [39] and 19,173 [40]. Studies were undertaken in a broad range of developed and developing countries, with 19 (35%) conducted in the USA. Study participants were most frequently children from the early year levels of school (i.e., pre-kindergarten to year 2), with 44 studies (80%) reporting outcomes for academic performance and motor proficiency in children at these year levels. Only seven (13%) studies [41,42,43,44,45,46,47] involved school students in high school (i.e., year 7 to 12). Socioeconomic status (SES) of study participants varied, with several studies undertaken specifically in socio-economically disadvantaged communities [48,49,50,51,52,53,54]. As outlined in Tables S1 and S2, a range of instruments were used to evaluate the different components of fine and gross motor proficiency. Examples of these instruments included standardized motor assessments, standardized developmental assessments, and individual motor subtests. A wide variety of instruments were also used to evaluate academic performance in mathematics and reading (Tables S1 and S2), often chosen depending upon the country in which the study was undertaken. Examples of these instruments included standardized academic achievement tests, national standardized assessments, teacher-reported academic reports, and grade point average (GPA). It is it is important to acknowledge that a wide range of covariates were also measured in the studies included in this review (Tables S1 and S2) and factored into their subsequent statistical analyses. For example, executive function (EF) was one of the key cognitive variables that was consistently reported in the included studies as a covariate. Components of EF that were assessed included working memory, cognitive flexibility (e.g., planning, problem solving, reasoning), inhibitory control, attention and behavioural self-regulation. Examples of additional covariates measured included age, gender, intelligence, visual perception, other academic variables (e.g., vocabulary, writing, spelling), family characteristics (e.g., SES, parental education, ethnicity), behavioural characteristics (e.g., social behaviour, classroom engagement), and physical characteristics (e.g., body mass index, PA levels, CRF, pubertal status).

### 3.2. Methodological Quality of Included Studies

The critical appraisal percentage (CAP) for each of the studies is shown in Tables S1 and S2. The level of agreement established between the two reviewers (K.M., N.M.) in their assessments of methodological quality was considered ‘substantial agreement’, based on the Cohen’s kappa analysis (k = 0.758) [34]. The mean (±SD) methodological quality score of the 51 observational studies in the review was 12.80 (±3.21) out of a possible 20 points, equating to a CAP of 64.02% (±16.06), with a CAP range of 20–90% (Table S1). A total of 16 (31%) of the observational studies were categorized as having ‘good’ methodological quality, 22 (43%) as being of “fair” methodological quality, and 13 (25%) as having ‘poor’ methodological quality. According to the five subcategories of the modified Downs and Black checklist, collectively across the observational studies the most notable limitations were in external and internal validity (both bias and confounding). For example, many studies included samples that were not considered representative of the population, limiting the ability to generalize findings to other populations. There were also deficits in reporting of the distributions of principle confounders in each group, as well as reporting of actual probability values for the main outcomes. Very few studies were adequately powered, or provided the basis for the study sample. Notable strengths of the studies were in the reporting quality category, meaning that the studies commonly provided descriptions of the study aim, main outcomes to be measured and participant inclusion and exclusion criteria, and used appropriate statistical tests to assess the main outcomes.

The mean (±SD) methodological quality score of the four experimental studies included in the review was 14 (±5.35) out of a possible 28 points, equating to a CAP of 50% (±19.12), with a CAP range of 25–71% (Table S2). One (25%) study was classified as being of ‘good’ methodological quality, one (25%) as being of ‘fair’ methodological quality, and the remaining two (50%) as having ‘poor’ methodological quality. The most notable limitations across the experimental studies were in the categories of external and internal validity, particularly confounding (selection bias) as well as a lack of sufficient power to detect a clinically important effect. For example, limitations in the reporting category included a lack of reporting of participant details, distributions of principle confounders in each group, adverse events, and characteristics of patients lost to follow up. In relation to external and internal validity, there were also limitations in representative sampling, blinding of subjects to the intervention, blinding of those measuring the primary outcomes, randomization into groups, concealment of randomized intervention, and reporting of whether participants lost to follow up were considered.

### 3.3. Aim 1: Relationships between Motor Proficiency and Academic Performance in Mathematics and Reading

#### 3.3.1. Fine Motor Proficiency and Academic Performance in Mathematics

A total of 29 (57%) of the observational studies (14 longitudinal and 15 cross-sectional) from the present review investigated the relationship between fine motor proficiency and academic performance in mathematics. Most studies (86%) examining these variables reported findings from participants in pre-kindergarten to year 2, with only three (11%) studies [43,44,45] reporting findings from participants in high school. A total of 19 (65%) studies [21,40,44,48,49,52,53,55,56,57,58,59,60,61,62,63,64,65,66] used standardized assessment tools to measure both fine motor proficiency and academic performance in mathematics. Of the 29 studies that examined these variables, 12 (41%) [40,48,51,54,56,57,59,64,65,66,67,68] were categorized as having ‘good’ methodological quality, 10 (34%) [44,49,52,55,58,60,61,62,69,70] were categorized as having ‘fair’ methodological quality, and seven (24%) [21,43,45,53,63,71,72] were categorized as having ‘poor’ methodological quality. A summary of the associations between the components of fine motor proficiency and academic performance in mathematics, along with the levels of evidence supporting the associations can be found in Table 1.

A total of five studies [45,53,55,56,66] examined the relationship between fine motor precision and academic performance in mathematics. Significant very weak to moderate positive correlations (r = 0.13−0.597) between fine motor precision and mathematics performance variables were reported by four studies [45,53,56,66], however only two of the studies were classified as having fair to good methodological quality [56,66]. A longitudinal study by Kim et al. [56] found several significant positive partial correlations (controlling for age) between fine motor coordination and mathematical skills for kindergarten and grade 1 cohorts at different measurement points across the study. However, several non-significant associations between fine motor coordination and mathematical skills were also reported for the cohorts at other measurement points. Kim et al. [56] concluded from their analyses that fine motor coordination seemed to contribute to mathematics performance indirectly, through visual motor integration, across kindergarten and grade 1 [56]. Another study [55] also reported non-significant associations between a draw-a-person task and applied problems subtest in kindergarten children. Overall summary coding suggests that despite inconsistent findings, there was evidence to support a significant very weak to moderate positive association between fine motor precision and academic performance in mathematics (see Table 1).

When taking into consideration all studies that examined associations between fine motor integration (also referred to as visual motor integration or copying skills) and mathematics performance variables (Table S1), 15 of the 16 studies found significant positive associations, with 12 of these significant associations reported by studies with fair to good methodological quality [48,49,52,54,55,56,57,58,60,62,64,69]. In studies analysing correlations between fine motor integration and mathematics performance, six studies [48,52,55,57,58,63] reported significant very weak to weak associations (r = 0.16−0.38), seven studies [49,53,56,58,60,64,71] reported moderate associations (r = 0.417−0.59), and two studies [56,64] reported a strong association (r = 0.612−0.673). The one study [45] that did not report a significant association between fine motor integration and mathematics performance was the only study conducted with high school participants. Overall, summary coding suggests there was a strong level of evidence to support a significant very weak to strong positive association between fine motor integration and academic performance in mathematics (see Table 1).

The relationship between manual dexterity and academic performance in mathematics was examined by nine studies [43,44,45,48,52,55,62,66,68]. Five of the six studies [43,48,52,55,66,68] reporting significant positive associations were classified as having fair to good methodological quality. The four studies [48,52,55,66] that reported results from correlation analyses found significant very weak to weak positive correlations (r = 0.11−0.37) between manual dexterity tasks and mathematical ability. One longitudinal study [68] found that the smaller number of cubes moved in a box and block task was associated with poorer arithmetic skills for boys in grades 1 and 2, but not for girls in the same grades. Another study [43], classified as having ‘poor’ methodological quality, reported significant strong correlations between the time taken to stack a tower of cubes and scores on a mathematics skills test for children aged nine to 16 years (r = −0.643 to −0.727, *p* < 0.05).

Two other studies [44,45] conducted with participants in high school did not report significant associations between manual dexterity and mathematics performance. Summary coding suggests that despite mixed findings, overall there was sufficient evidence to support a significant positive very weak to weak association between manual dexterity and mathematical skills (see Table 1).

A total of nine studies [21,40,51,55,59,65,67,70,72] examined associations between total fine motor scores and academic performance in mathematics. All studies reported significant positive associations with seven studies [40,51,55,59,65,67,70] classified as having fair to good methodological quality. Of note is that four studies [21,40,59,65] reported findings from the Early Childhood Longitudinal Study—Kindergarten Cohort and collectively found that fine motor skills were predictive of later mathematics achievement. Furthermore, five studies [55,59,67,70,72] reported results from correlational analyses and found significant very weak to strong positive associations (r = 0.25–0.73). Overall, summary coding suggests there was a strong level of evidence to support a significant very weak to strong positive relationship between total fine motor scores and mathematics performance (see Table 1).

#### 3.3.2. Gross Motor Proficiency and Academic Performance in Mathematics

A total of 24 (47%) observational studies (10 longitudinal and 14 cross-sectional) from the review investigated the relationship between gross motor proficiency and academic performance in mathematics. A summary of the data extracted from these studies can be found in Table S1. Findings from participants in pre-kindergarten to year 2 were reported in 14 (58%) studies, whereas seven (29%) studies [41,42,43,44,45,46,47] reported findings from participants in high school. Less than half (46%) of the studies [21,41,44,55,57,59,61,73,75,76,78] used standardized assessment tools to measure both gross motor proficiency and academic performance in mathematics. Of the 24 studies that examined these variables, eight (33%) [42,51,54,57,59,67,68,73] were categorized as having ‘good’ methodological quality, ten (42%) [44,46,55,61,69,70,74,75,76,78] were categorized as having ‘fair’ methodological quality, and six (25%) [21,41,43,45,47,77] were categorized as having ‘poor’ methodological quality. The overall levels of evidence from studies examining associations between gross motor proficiency and academic performance in mathematics in typically developing school-aged children and adolescents are outlined in Table 1.

It was apparent that associations between several components of gross motor proficiency, particularly bilateral coordination and strength, and mathematical performance had been examined less frequently by the studies included in the review, leading to more uncertain findings overall. The five studies [42,43,44,45,73] that examined associations between upper limb coordination and mathematical performance involved participants in school year levels 4–10. Significant very weak to moderate positive correlations (r = 0.13–0.439) between upper limb coordination tasks and mathematics performance were reported by four studies [42,43,44,73], with three of these studies [42,44,73] classified as having fair to good methodological quality. The two studies [42,45] that found non-significant associations assessed upper limb coordination through a dribbling task and used teacher-reported grades to assess mathematics performance. For example, one longitudinal study [42] reported conflicting findings, with significant very weak positive correlations (r = 0.18, *p* < 0.05) found between a dribbling task assessed in grade 7 and maths for boys only, with no significant associations found between these variables for girls. Overall, despite inconsistent findings, summary coding suggests there was sufficient evidence to support a significant very weak to moderate positive association between upper limb coordination and mathematical skills (see Table 1).

A total of five studies [44,45,68,74,75] analysed the relationship between balance and academic performance in mathematics. One study [74] assessed balance using a static single leg balance task, reporting several significant very weak to weak positive correlations (r = 0.26–0.37) between the balance task and mathematical performance. This same study [74] reported significant partial correlations (controlling for age, attentional and reasoning capabilities) between balance tasks with eyes closed and complex arithmetic tasks but non-significant partial correlations with more simple arithmetic tasks. Collectively, the three remaining studies [44,68,75], with fair to good methodological quality, found no significant association between balance and mathematics performance. Overall, summary coding suggests there was a sufficient level of evidence to support no significant relationship between balance and academic performance in mathematics (see Table 1).

Only three studies [45,69,75] examined associations between bilateral coordination and academic performance in mathematics. One study by Geertsen et al. [69] found that better performance in a gross motor task (i.e., a shorter time to complete a coordination wall task) was associated with better scores on a standardized mathematics test. Another study by Murrihy et al. [75] reported a significant weak positive correlation (r = 0.26, *p* < 0.05) between a finger-to-nose test and a standardized mathematics test. However, a study by Van Niekerk [45] reported conflicting results, with a significant weak positive correlation (r = 0.23, *p* < 0.05) found between a task involving tapping feet and fingers and teacher-reported maths results for boys and girls, but no significant association found between a jumping-in-place task (same sides synchronized) and teacher-reported maths results. Overall, summary coding suggests the level of evidence to support a significant relationship between bilateral coordination and mathematics was uncertain, due to a limited number of studies examining these variables (see Table 1).

A total of seven studies [42,45,46,68,73,75,76] examined the relationship between speed and agility and mathematical skills, with six studies [42,45,46,68,73,76] using the shuttle run to assess speed and agility. Only one study [45] was classified as having ‘poor’ methodological quality. Significant positive associations between speed and agility and academic performance in mathematics were reported by six studies [42,45,46,68,73,76]. In analysing correlations between speed and agility and mathematics variables, two studies [42,45] reported significant very weak to weak positive associations (r = 0.18–0.20). One longitudinal study [42] found significant very weak positive correlations (r = 0.18–0.20) between the 10 × 5 m shuttle run (assessed in grade 8) and marks in mathematics (assessed in grade 9) for both boys and girls. However, several non-significant correlations between these variables were reported at other measurement times in the study [42] for girls more often than boys. Another longitudinal study [68] reported a similar trend with significant findings between shuttle run test times and arithmetic skills reported more often for boys than girls in grades 1–3. Three other studies [68,73,76] reported that shuttle run test times were significantly but inversely associated with mathematics performance, with longer shuttle run test times related to poorer mathematics performance. Finally, one study [75] reported no significant association between performance on a jumping task and standardized maths test. Overall, summary coding suggests there was a strong level of evidence to support very weak-to-weak positive associations between speed and agility and mathematics performance (see Table 1).

Only two studies [45,76] with poor to fair methodological quality investigated the relationship between strength and mathematics performance, reporting significant very weak to weak positive associations (r = 0.15–0.29). The components of strength that were assessed in one of the studies included the sit up and standing broad jump from the European physical fitness test battery [76], while the other study assessed push ups and sit ups from the BOT-2 (Short Form) [45]. Overall, summary coding suggests the level of evidence to support a significant relationship between strength and mathematics performance was uncertain due to a limited number of studies examining these variables (see Table 1).

Associations between total gross motor scores and academic performance in mathematics were examined often, with 10 [42,47,55,57,59,67,68,70,77,78] of 13 studies reporting significant findings between these outcomes. Eight [42,55,57,59,67,68,70,78] of these 10 studies were classified as having fair to good methodological quality. Significant positive correlations reported in studies ranged from very weak to moderate (r = 0.16–0.41). Of the four studies that reported non-significant associations between total gross motor scores and mathematical performance, two [21,41] were classified as having ‘poor’ methodological quality. One longitudinal study [68] found significant positive associations between overall motor performance and arithmetic skills for boys in grades 1–3, but not for girls in grades 1 and 3. The findings from regression analyses reported in two other longitudinal studies [21,51] revealed that total gross motor scores (as measured by developmental motor assessments) were not a significant predictor of later mathematics ability. Another study [41] conducted with high school students found no significant associations between total gross motor coordination scores and a national standardized mathematics test. Overall, despite several conflicting findings, summary coding suggests there was a strong level of evidence to support a very weak to moderate positive association between total gross motor scores and mathematical skills (see Table 1).

Finally, a total of three studies [45,54,61] with poor to good methodological quality reported significant associations between total motor proficiency (a combination of fine and gross motor scores) and academic performance in mathematics using the BOT-2 (Short Form) to assess total motor proficiency. Significant positive correlations reported were found to be weak (r = 0.21–0.23). In summary, there was some evidence to support a significant relationship between total motor proficiency and academic performance in mathematics, however, summary coding suggests that overall the level of evidence for an association was uncertain due to a limited number of studies in the review examining these variables (see Table 1).

#### 3.3.3. Fine Motor Proficiency and Academic Performance in Reading 

A total of 30 (58%) observational studies (16 longitudinal and 14 cross-sectional) from the present review investigated the relationship between fine motor proficiency and academic performance in reading. A summary of the data extracted from these studies can be found in Table S1. Findings from participants in pre-kindergarten to year 2 were reported in the majority (90%) of studies, with only one study examining these variables in participants in high school [44]. Standardized assessment tools were used to measure both fine motor proficiency and academic performance in reading for 24 (80%) of the studies. Of the 30 studies that examined these variables, nine (30%) [40,48,54,57,59,66,68,79,80] were categorized as having ‘good’ methodological quality, 14 (47%) [44,52,55,58,60,61,62,69,81,82,83,84,85,86] were categorized as having ‘fair’ methodological quality, and seven (23%) [21,39,53,63,71,87,88] were categorized as having ‘poor’ methodological quality. A summary of the overall levels of evidence from the studies examining the associations between the components of fine motor proficiency and academic performance in reading can be found in Table 2.

A total of four studies [53,55,66,86] examined associations between fine motor precision and academic performance in reading for children in pre-kindergarten to year 1. Three studies [55,66,86] with fair to good methodological quality reported significant positive associations between fine motor precision and reading variables, with the strength of the correlation classified as very weak to weak (r = 0.15–0.28). However, one study [53] reported no significant associations between the fine motor precision subtest from the BOT-2 and a word reading subtest from a standardized reading test. In summary, while there was some evidence to support a significant positive association between fine motor precision and reading performance, overall, summary coding suggests the level of evidence for an association was uncertain due to a limited number of studies examining these outcomes (see Table 2).

The relationship between fine motor integration skills and academic performance in reading was examined most often, with 17 [39,48,52,53,54,55,58,60,63,69,71,81,83,85,86,87,88] significant positive associations out of the 22 reported. In relation to the strength of correlations between variables reported, 10 studies [48,52,53,55,58,63,81,83,85,87] found very weak to weak correlations (r = 0.163–0.38), six studies [39,58,59,71,81,88] found moderate associations (r = 0.40–0.47), and two studies [60,71] reported a strong correlation (r = 0.60–0.62). A total of 11 [48,52,54,55,58,60,69,81,83,85,86] of the 17 studies reporting significant associations had fair-to-good methodological quality. Two studies [39,85] reported mixed findings with significant positive associations found between visual motor integration tasks and certain constructs of reading performance but non-significant findings for other constructs of reading (Table S1). Eight studies [39,53,57,62,63,79,83,85] reported that when other known predictors of reading (e.g., intelligence quotient (IQ), vocabulary, phonological awareness) were included in regression analyses, fine motor integration was not found to contribute significantly to predicting reading achievement. However, four [39,53,83,85] of the eight studies reported significant correlations between fine motor integration and reading performance prior to accounting for these covariates. In summary, despite several inconsistencies reported between studies, summary coding suggests there was a strong level of evidence to support a significant very weak to strong positive relationship between fine motor integration and reading performance (see Table 2).

A total of seven [48,52,55,66,68,82,86] studies with fair to good methodological quality reported significant positive associations between manual dexterity and academic performance in reading. The level of correlation reported ranged from very weak to weak (r = 0.15–0.36). One study [68] reported mixed results for associations between a box and block test and two reading variables, with significant associations found for reading fluency, particularly for boys, but non-significant associations reported for reading comprehension. Furthermore, six studies [44,52,62,66,82,86] conducted regression analyses and found that manual dexterity performance did not make a unique contribution to reading performance in the presence of other predictors (e.g., executive function, phonological awareness). However, four [52,66,82,86] of these studies reported significant positive correlations between manual dexterity and reading performance. Overall, summary coding suggests the level of evidence to support a relationship between manual dexterity and reading performance was inconsistent as less than 60% of studies supported this relationship (see Table 2).

Finally, all six studies [21,40,55,59,67,86] that examined the relationship between total fine motor scores and reading performance reported significant positive associations. Fair to good methodological quality was found for five [40,55,59,67,86] of the six studies. A study by Suggate et al. [86] found mixed results, reporting significant very weak to weak positive correlations (r = 0.18–0.23) between the total score of the manual dexterity subtest of the Movement Assessment Battery for Children (MABC) and reading outcomes (specifically phonemic awareness and word reading), however, associations between manual dexterity and a letter naming subtest were not significant. Conversely, three studies [21,40,59] reported findings from the Early Childhood Longitudinal Study—Kindergarten Cohort (ECLS-K) and collectively found that fine motor skills were positively associated with and a very strong and consistent predictor of later achievement in reading. In the ECLS-K study [21,40,59], the Early Screening Inventory—Revised was used to assess performance on seven fine motor tasks. Overall, summary coding suggests there was a strong level of evidence to support a significant very weak-to-weak positive association between total fine motor scores and reading performance (see Table 2).

#### 3.3.4. Gross Motor Proficiency and Academic Performance in Reading

A total of 21 (41%) studies (11 longitudinal and 10 cross-sectional) from the present review investigated the relationship between gross motor proficiency and academic performance in reading. A summary of the data extracted from these studies can be found in Table S1. Findings from participants in pre-kindergarten to year 2 were reported in 14 (67%) studies, and four (19%) studies [41,42,44,47] examined these variables in participants in high school. A total of 14 (67%) studies [21,41,44,55,57,59,61,73,75,76,78,80,84,89] used standardized assessment tools to measure both gross motor proficiency and academic performance in reading. Of the 21 studies that examined these variables, eight (38%) [42,54,57,59,67,68,73,80] were categorized as having ‘good’ methodological quality, nine (43%) [44,55,61,69,75,76,78,84,89] were categorized as having ‘fair’ methodological quality, and four (19%) [21,41,47,87] were categorized as having ‘poor’ methodological quality. A summary of the overall levels of evidence from the studies examining the associations between the components of gross motor proficiency and academic performance in reading can be found in Table 2.

A total of four studies [42,44,73,89] with fair to good methodological quality reported significant very weak to weak positive correlations (r = 0.10–0.28) between upper limb coordination and reading skills. A study by Aadland et al. [73] found significant associations between the catching subtest from the Movement Assessment Battery for Children (2nd Edition) (MABC-2) and results on a standardized reading test but non-significant associations between the aiming subtest from the MABC-2 and reading performance. Mixed results were also reported in another study [42] where significant very weak positive correlations (r = 0.17, *p* < 0.05) were found between a dribbling task and marks in Finnish language for girls in grade 7 but not for boys. Overall, despite some inconsistencies in findings, summary coding suggests there was a strong level of evidence to support a significant weak positive association between upper limb coordination and reading performance (see Table 2).

Associations between balance and academic performance in reading were examined by four studies [44,68,75,87] that assessed balance using different instruments. One study [87] reported significant very weak to weak associations (r = −0.117 to −0.251) between the inclination from upright measured in a postural stability task and performance on a reading task. The other three studies [44,68,75], classified as having fair to good methodological quality, found no significant associations between balance and reading performance, except for the study by Haapala et al. [68] that found that poor performance on a static single leg balance test was related to poor reading comprehension for boys in grade 1. In summary, there was some evidence that no significant association exists between balance and academic performance in reading, however, summary coding suggests that overall the level of evidence was uncertain due to an insufficient number of studies in the review examining these variables (see Table 2).

Only two studies [69,75] categorized as having ‘fair’ methodological quality examined the association between bilateral coordination and reading performance, both reporting significant positive associations between coordination tasks and standardized reading tests. A study by Murrihy et al. [75] reported the strength of the correlation between a finger-to-nose task and letter–word identification subtest was weak (r = 0.33, *p* < 0.001). Another study [69] found that a shorter time to complete a wall coordination task was associated with better scores in a standardized reading test. In summary, there was some evidence to support a significant relationship between bilateral coordination and reading performance, however, overall summary coding suggests the level of evidence was uncertain due to a limited number of studies in the review examining these variables (see Table 2).

Associations between speed and agility and reading performance were examined by six studies [42,68,73,75,76,89]. The four studies [42,68,73,89] that reported significant positive associations had fair to good methodological quality. Two studies [42,89] reported significant very weak to weak positive correlations (r = 0.16–0.31) between locomotor skills (e.g., leaping, hopping) and reading outcomes. Another two studies [68,73] reported that shuttle run test times were significantly but inversely associated with reading performance; however in one study this applied only to boys in grades 1–3. These findings were consistent with those reported by Jaakkola et al. [42] who reported significant correlations between a 5 × 10 m shuttle run test and marks in Finnish language for boys in grades 7 and 8 but found non-significant correlations for girls. Another study [76] did not find a significant relationship between the time taken to perform a 5 × 10 m shuttle run and a standardized reading test in participants aged 7–12 years. In summary, there appears to be some inconsistency in the findings reported in the studies examining the associations between speed and agility and reading performance, potentially due to the instrument used to measure speed and agility (e.g., assessment of locomotor skills vs shuttle run). Therefore, despite there being some evidence to support a significant association between speed and agility and academic performance in reading, overall summary coding suggests the level of evidence was inconsistent as less than 60% of studies supported a significant relationship (see Table 2).

Mixed findings were reported in the only study [76] that examined the relationship between strength and reading performance. For example, a significant very weak positive association (r = 0.18, *p* < 0.01) was found between distance measured on the standing broad jump and scores on a standardized reading test; however, no significant association was found between the number of sit-ups performed and the same standardized reading test. Overall, summary coding suggests the level of evidence to support a significant association between strength and reading performance was uncertain, due to a limited number of studies examining these variables (see Table 2).

A total of 12 studies [21,41,42,47,55,57,59,67,68,78,84,89] examined the relationship between total gross motor scores and academic performance in reading with eight studies using standardized tests to assess both outcomes. Eight studies [42,55,57,59,67,78,84,89] reported significant very weak to moderate positive correlations (r = 0.15–0.404). Another study [68] also found that poor overall motor performance was associated with worse academic results in reading fluency and reading comprehension. All nine studies reporting significant associations had fair to good methodological quality. However, there were inconsistencies in the findings reported within three studies [42,55,78], often dependent on gender or the academic variable being assessed. Two studies [78,84] found significant positive associations between gross motor composite scores and reading performance in 9-10-year-old girls only. Another study [42] reported significant very weak to weak correlations (r = 0.17–0.23) between marks in Finnish language and total scores for fundamental movement skills for boys in grades 7–9 but found non-significant associations for girls. A study by Cameron et al. [55] reported several significant very weak to weak correlations (r = 0.17–0.20) between gross motor composite scores from a developmental assessment and reading composite scores, but non-significant associations between gross motor composite scores and results on individual reading subtests, assessed at a different time. Finally, four studies [21,55,57,78] reported that following regression analyses, total gross motor scores did not make a unique contribution to reading performance in the presence of other predictors. In summary, despite several inconsistencies reported between studies, overall summary coding suggests there was a strong level of evidence to support a significant very weak to moderate positive association between total gross motor scores and academic performance in reading (see Table 2).

Finally, four studies [54,61,80,84] classified as having fair to good methodological quality, examined associations between total motor proficiency (combined fine and gross motor scores) and academic performance in reading. Total fine and gross motor scores were assessed using the MABC [80,84] and the BOT-2 (Short Form) [54,61]. A study by Pienaar et al. [54] reported a strong positive relationship between total motor proficiency and academic performance in reading. However, a study by McPhillips et al. [80] found that although motor skills were weakly predictive of reading without confounders, they were not predictive of reading in the context of other predictors. A study by Cadoret et al. [61] reported a significant weak positive correlation (r = 0.28, *p* < 0.01) between total motor proficiency and academic achievement in reading; however, a structural equation modelling analysis found that the mechanism appeared to be through an indirect path, via cognitive ability. Another study [84] found no significant association between overall motor competence and a reading achievement test in children aged 9–10 years. In summary, there was some evidence to support a significant relationship between total motor proficiency and reading performance, however, summary coding suggests that overall the level of evidence for an association was uncertain due to a limited number of studies in the review examining these variables (see Table 2).

### 3.4. Aim 2: Impact of Motor Proficiency-Related Interventions on Academic Performance in Mathematics and/or Reading

A total of four experimental studies [50,90,91,92] investigating the impact of motor proficiency-related interventions on academic performance in mathematics and reading of school-aged children were eligible for inclusion in the review (Table S2). A cluster randomized study by Beck et al. [90] investigated whether fine or gross motor activity integrated into mathematics lessons over a 6-week period could improve children’s mathematical performance. A quasi-experimental study by Callcott et al. [91] investigated whether pre-kindergarten children who participated in a year-long program involving literacy and movement would demonstrate superior results in measures of movement and early literacy skills when compared with students receiving a literacy only intervention, movement only intervention or no intervention (control group). A further quasi-experimental study by Erasmus et al. [50] aimed to establish the effect of a 10-week perceptual–motor intervention programme on school readiness (including assessment of a number concept subtest) of children in pre-kindergarten. Finally, another quasi-experimental study by Ericsson [92] measured whether daily PE and motor training over a 3-year period would impact attention and school results in reading and mathematics.

The interventions described in each study were delivered in the primary school setting, with participants in the early year levels of schools (pre-kindergarten to Year 2). Interventions ranged in duration from 6 weeks [90] to 3 years [92]. The interventions described in each study involved implementing motor skills into a prescribed number of lessons each week for a specified timeframe. Intervention parameters, including the type, duration and frequency of the intervention varied across studies. For example, the intervention described by Beck et al. [90] involved the implementation of fine or gross motor activities into a 60-min mathematics lesson, three days a week over a 6-week period (Table S2). Two interventions were delivered by the classroom teacher [90,91], one intervention was delivered by the researcher [50], and one intervention was delivered by the PE teachers and representatives from local sports clubs [92]. Two studies [90,91] described their strategies to enhance compliance with the intervention, with these strategies including the provision of professional development workshops to classroom teachers, to teach them how to implement the intervention, along with follow up support throughout the intervention period.

All four experimental studies [50,90,91,92] reported a statistically significant effect of the motor skill intervention on academic performance in mathematics and/or reading. Two experimental studies [50,90] in the review incorporated fine motor skills into their interventions to examine their effect on academic performance. In their cluster randomized control trial, Beck et al. [90] reported that participants in the fine motor-enriched learning group, particularly those with normal mathematics performance, improved their performance on the mathematics task following the 6-week intervention. Statistical analyses were conducted to determine the specific impact of fine motor enriched learning activities on mathematical performance, with Beck et al. [90] reporting that changes in fine motor skill performance accounted for approximately 10.7% of the effects of the intervention on mathematics performance. The intervention outlined in the study by Erasmus et al. [50] involved the provision of a 40-min lesson incorporating fine motor, gross motor, and perceptual motor skills, three days per week over 10 weeks. Participation in the intervention led to a significant improvement in results on the number concept subtest of a standardized developmental assessment (*p* < 0.012, Cohen’s effect size d = 1.13). However, improvements in the number concept subtest were not significantly better than the control group following the intervention (controlling for differences in pre-test scores) [50].

Each of the four experimental studies in the review incorporated gross motor skills into their interventions to examine their effect on academic performance [50,90,91,92]. Beck et al. [90] incorporated gross motor-enriched learning activities into mathematics lessons, leading to greater improvements in mathematical performance compared to the conventional (control) group and fine motor enriched learning group, particularly in students with normal mathematics performance. Beck et al. [90] reported that changes in gross motor skill performance accounted for approximately 25% of the effects of the intervention on mathematics performance. However, there were no differences in mathematics performance reported between groups when re-assessed 8 weeks after the intervention [90]. A quasi-experimental study by Callcott et al. [91] found that incorporating a combination of movement (i.e., 15 min of action songs) and literacy skills (15 min of phonological awareness and decoding activities) into daily lessons led to students performing significantly better on reading measures (phonological awareness) than students in the literacy only, movement only and conventional (control) groups. As previously mentioned, following the 10-week motor skills intervention outlined by Erasmus et al. [50] the experimental group significantly improved their mathematical skills (number concept) but not significantly more than the control group. Finally, the quasi-experimental study by Ericsson [92] found that participation in daily lessons of PE and motor training led to students in the intervention group achieving better results than those in the control group in national tests for reading (overall large difference in results between groups, Cramer’s index 0.29) and for mathematics (overall small difference in results between groups, Cramer’s index 0.21).

In summary, a limited number of experimental studies with varied study designs, intervention parameters, and methodological quality have examined whether motor proficiency-related interventions impact academic performance in mathematics and reading. However, there were findings of a statistically significant effect of the motor skill interventions on academic performance that warrants further investigation.

## 4. Discussion

### 4.1. Overview of Findings

The overall objective of this systematic review was to identify, critically appraise, and synthesize the findings of studies examining the relationship between motor proficiency and academic performance in mathematics and reading in typically developing school-aged children and adolescents.

To address the first aim of the review, 51 observational studies were examined to determine whether there was evidence for significant associations between components of motor proficiency and academic performance in mathematics and reading. In summary, based on the findings from observational studies, of which 74% were classified as having fair to good methodological quality, there was sufficient evidence to support significant very weak to strong positive associations between all components of fine motor proficiency and academic performance in mathematics. Although fewer studies in the review examined the relationship between the components of gross motor proficiency and academic performance in mathematics, sufficient evidence also emerged to support significant very weak to weak positive associations between gross motor proficiency (specifically the components of upper limb coordination, speed and agility, and total gross motor scores) and academic performance in mathematics. There was also sufficient evidence to support no significant association between balance and mathematics performance. A similar trend of significant associations was evident in studies examining the relationship between motor proficiency and academic performance in reading, although there were more inconsistencies reported between studies. Overall, there was evidence to support a significant very weak to strong relationship between fine motor proficiency (specifically the components of fine motor integration and total fine motor scores) and academic performance in reading. There was also sufficient evidence to support a significant very weak to weak positive association between academic performance in reading and upper limb coordination, as well as total gross motor scores.

To address the second aim of the review, the findings from four experimental studies were synthesized to determine whether motor proficiency-related interventions impact academic performance in mathematics and/or reading in typically developing school-aged children and adolescents. Based on the findings from one cluster randomized controlled trial and three quasi-experimental studies, there was evidence for a statistically significant effect of the motor skill intervention on academic performance compared to the control group in each study for students in the early years of school (pre-kindergarten to year 2). However, several methodological limitations relating to the external and internal validity (bias and confounding) of studies were apparent; thus, it is difficult to infer the exact underlying mechanisms for the effects of the interventions, and results should be interpreted with caution.

### 4.2. Relationships between Motor Proficiency and Academic Performance

#### 4.2.1. Fine Motor Proficiency and Academic Performance

There was consistency in findings among studies included in the review that a relationship exists between motor proficiency and academic performance in mathematics and reading; however, this association differed between the components of fine and gross motor proficiency. There was evidence from observational studies for significant positive associations between the majority of components of fine motor proficiency and academic performance in mathematics and reading. This was with the exception of the components of fine motor precision and manual dexterity and their associations with reading performance as there was either an insufficient number of studies in the review examining these variables or inconsistent evidence.

Differences in associations between motor proficiency and academic variables for children and adolescents were also apparent, consistent with those reported by van der Fels et al. [22]. Interestingly, the majority (86%) of observational studies in the present review that reported significant positive associations between the components of fine motor proficiency and academic performance involved study participants in the early year levels of school (pre-kindergarten to year 2). In particular, relationships between fine motor integration (visual motor integration) and academic performance in mathematics and reading were examined most often in this age group, with a strong level of evidence found to support very weak to strong positive associations between these variables. Thus, findings from this review may have important implications for this age group upon entry to school. Only seven studies in the present review involved high school students and consequently there was insufficient evidence to support associations between fine motor proficiency and academic performance in mathematics and reading in this older age group. However, non-significant associations in adolescents have been proposed by the authors of some studies by a potential ‘ceiling effect’ that occurs as primary school students achieve automaticity with their fine motor precision and manual dexterity skills [65].

It is worth noting that many studies examining the relationship between fine motor proficiency and academic performance in mathematics and reading in the present review conducted statistical analyses that accounted for other covariates, in addition to correlational analyses. For example, regression analyses accounting for key cognitive confounders (e.g., visual–spatial skills, executive function) were performed in several studies investigating the relationship between fine motor proficiency and mathematics and found that fine motor integration remained independently predictive of academic performance in mathematics. However, when examining the relationship between fine motor proficiency and reading performance, it was often reported that in the presence of other predictors (e.g., IQ, executive function, phonological awareness), fine motor integration and manual dexterity did not uniquely contribute to reading performance [57,62,83,85]. The underlying mechanisms to potentially explain the difference in findings between the components of fine motor proficiency and these two core academic areas is beyond the scope of the present review; however, it has been proposed there may be both biological and learning mechanisms that underpin these relationships [93,94]. Furthermore, it suggests that future studies should ensure that covariates known to impact on the relationship between motor proficiency and academic performance, such as age, gender, SES, executive function (and its components), body mass index, and CRF, are measured and reported on when examining these outcomes in school-aged children and adolescents.

#### 4.2.2. Gross Motor Proficiency and Academic Performance

The relationships between the components of gross motor proficiency and academic performance in mathematics and reading have been investigated less frequently in the literature. For example, four or less studies in the review investigated associations between academic performance in mathematics and reading and the components bilateral coordination and strength, along with total motor proficiency scores. Consequently, the evidence to support associations between these variables was uncertain, despite several significant positive associations reported by the studies examining them. There was sufficient evidence to suggest there was no association between balance and academic performance in mathematics, with similar trends reported for reading, though this was based on findings from a limited number of studies. However, there was sufficient evidence to support a significant very weak to weak positive association between academic performance in mathematics and reading and upper limb coordination, with promising findings also found for speed and agility. Interestingly, these relationships were assessed in several studies involving high school participants. This warrants further investigation to determine whether more complex gross motor skill training may impact the academic performance of students in both primary and high school.

Several findings from the present review appear to be consistent with those reported in the systematic review by van der Fels et al. [22] in that the authors also found evidence to support a weak to moderate relationship between academic skills and object control skills. However, in contrast to the findings in the present review, van der Fels et al. [22] reported there was insufficient evidence to support a relationship between academic skills and fine and gross motor skills and they found no evidence to support a relationship between academic skills and bilateral body coordination and timed performance in movements. This contrast in findings from the present review is not surprising, given the limited number of studies that specifically examined the relationship between academic skills and motor skills that were eligible for inclusion in the review by van der Fels [22]. Therefore, the findings from the present review can contribute significantly in synthesizing the rapidly expanding body of literature examining the relationship between motor proficiency and academic performance in mathematics and reading in typically developing school-aged children and adolescents.

### 4.3. Impact of Motor Proficiency Interventions on Academic Performance

Collectively, the findings from the four experimental studies included in the review provide results of a statistically significant effect of motor skill interventions on the mathematics and reading performance of children, particularly in the early years of school. These results add to the growing body of literature investigating the impact of classroom-based PA on the educational outcomes of school students. However, to date, studies on this topic have generally been unable to consistently provide sufficient detail regarding whether the type of PA used in classroom-based interventions involves aerobic, motor, or strength-based activities, or a combination of all types of PA [25]. This information is essential in understanding the exact type, frequency, duration, and intensity of PA used in classroom-based PA interventions that may be required to impact learning [8,25]. Therefore, the present review included studies that specifically incorporated motor skill interventions into the regular school day with studies evaluating both academic performance and motor proficiency outcomes before and after the interventions.

The interventions outlined in the studies by Beck et al. [90] and Callcott et al. [91] involved the integration of movement into mathematics and literacy lessons, respectively, thus providing examples of classroom-based PA with an academic focus specifically incorporating motor skills as the type of PA. The positive findings from these two studies included in the present review are generally consistent with those reported by several recently published systematic reviews [8,13,23,24]. In their review, Donnelly et al. [8] reported mixed findings from five studies investigating the impact of integrating PA into academic lessons (or physically active lessons) on academic achievement. However, three of the five studies found positive associations for increased academic achievement in mathematics following physically active lessons [8]. A recently published systematic review and meta-analysis by Watson et al. [24] evaluated the impact of classroom-based PA interventions on academic-related outcomes reported in 39 studies. A total of seven studies from the review assessed the effect of physically active lessons on mathematics achievement, with four of the seven studies reporting positive outcomes [24]. A total of five studies assessed the effect of physically active lessons on reading performance, with three of the five studies reporting improved reading performance in the intervention group. Overall, the authors from both reviews [8,24] concluded that despite some inconsistent findings, the integration of PA into academic lessons generally appeared to have a positive impact on academic performance.

Classroom-based PA can also involve incorporating PA into the regular school day routine, without an academic focus. In the present review, the intervention described in the study by Erasmus et al. [50] involved a 10-week program delivered by the researcher that involved incorporating fine, gross, and perceptual motor exercises into the regular school day; however, the program did not appear to have a specific academic focus. In their review, Donnelly et al. [8] reported that two studies examined the impact of specialized PA programs in the school setting on academic performance, such as the one described by Erasmus et al. [50], with both studies also reporting significant improvements in academic performance in mathematics and reading.

The intervention outlined in the study by Ericsson [92] in the present review involved the addition of three extra PE classes into the school week, along with one extra motor training class. The positive findings in this study are in contrast to those reported in the review by Donnelly et al. [8] who found that only two of six studies reported a positive effect on academic achievement scores when implementing additional or enhanced PE into the school day. However, it is worth noting that the studies included in the review by Donnelly et al. [8] used cluster randomized designs whereas the study by Ericsson [92] was classified as having ‘poor’ methodological quality.

Collectively the authors from reviews on this topic [8,13,23,24] highlighted that the implementation of classroom-based PA into the school environment is still a relatively new concept and thus the methodological quality of intervention studies is varied. Several limitations within experimental study designs were noted, including variation in study design, intervention content, and outcome assessment [8,24]. In addition, few studies reported the theoretical rationale behind the intervention and provided sufficient details about the intervention, including examples of intervention sessions. This is consistent with the present review where a varying level of methodological quality was also apparent in the four experimental studies, along with varied intervention parameters. This suggests that further research is required using more robust study designs, to explore further the impact of integrating motor skills into academic lessons and/or the regular school day.

### 4.4. Strengths and Limitations

The present review had several key strengths. Firstly, this review systematically synthesized the findings of 55 peer-reviewed studies including a large sample of typically developing school-aged participants from over 20 different countries. Secondly, to minimize reporting bias, a comprehensive search of health and education databases was conducted in accordance with the PRISMA guidelines [26], followed by a systematic screening approach to identify eligible studies. Thirdly, to address the first aim of the study, a thorough appraisal of the methodological quality of each study was undertaken using the modified Downs and Black tool [30] that helped guide the interpretation of levels of evidence reported by cross-sectional and longitudinal studies for associations between motor proficiency and academic performance variables. Finally, this review allowed for a more in-depth examination of associations between the individual components of performance-related physical fitness (motor proficiency in this instance) and two core academic areas at school (mathematics and reading) than previously reported. Understanding which specific components of fine and gross motor proficiency are more strongly related to mathematical and reading skills may inform the design of future experimental studies to further ascertain whether a cause and effect relationship exists.

However, it is important to acknowledge there were also several key limitations in this review. Firstly, a limited number of experimental studies with robust study designs (e.g., randomized controlled trials) were eligible for inclusion in the present review, with findings synthesized from predominantly cross-sectional, longitudinal studies and quasi-experimental designs. Secondly, there was considerable heterogeneity of the outcome measures used between studies to assess motor proficiency and academic performance in mathematics and reading, making it difficult to clearly compare and interpret the findings across studies.

The approach used to classify different motor skills measured by studies into components of motor proficiency may also be a potential limitation in the current review. For example, it was the authors’ discretion to determine the most appropriate category of motor proficiency for each motor skill measured, based on Bruininks’ definitions of motor proficiency subtests [27]. Additionally, outcomes for academic performance in mathematics and reading were categorized broadly in the present review for ease of interpretation, in favour of being classified by their underlying constructs. Therefore, we were unable to determine which specific constructs of mathematics and reading may be more strongly related to each component of motor proficiency.

The search strategy in this review was limited to including studies specifically examining associations between motor proficiency and academic performance in mathematics and reading. However, it was evident that numerous covariates may also impact the findings reported in studies when examining typically developing school-aged children and adolescents. These covariates included demographic factors (e.g., age, gender, SES), cognitive factors (e.g., executive function and its components), and physical factors (e.g., body mass index, PA levels, CRF, and other health-related fitness measures). Although the covariates reported by each eligible study were extracted and recorded, their potential contribution to the overall findings were not discussed in detail as this was beyond the scope of the review.

There was variation in the parameters for the motor skill interventions reported among the four experimental studies included in the review; thus, factors such as type and intensity of motor skill training, along with the duration and frequency of lessons per week, differed in each study, making it difficult to determine the most effective dose. Given the strict inclusion/exclusion criteria for the present review, several experimental studies reporting motor skill interventions were ineligible for inclusion in the review. These studies were ineligible as they did not report findings for academic performance in mathematics or reading separately, often combining scores of multiple academic areas to provide an overall academic achievement score [95,96]. Other experimental studies were ineligible as they reported an overall fitness score (e.g., a combination of health and performance-related fitness) [97] or they did not assess motor proficiency at all [98,99]. The collective findings reported by these five experimental studies [95,96,97,98,99], ineligible for inclusion in the present review, also found a positive impact of motor skill programs delivered in the school setting on academic performance. Finally, the findings from experimental studies synthesized in the present review are based on the published evidence available, and thus, publication bias may potentially exist if several other studies that have not found significant findings remain unpublished.

## 5. Conclusions

This systematic review adds considerably to the rapidly expanding body of literature examining associations between PA, fitness, cognition, and academic performance. The present review found evidence to support significant positive associations between several components of motor proficiency and measures of academic performance in mathematics and reading. There was evidence that all components of fine motor proficiency were significantly and positively associated with academic performance in mathematics, particularly during the early years of school. Similar evidence was found to support a significant positive relationship between fine motor proficiency (specifically fine motor integration and total fine motor scores) and academic performance in reading. There was also evidence for significant positive associations between academic performance in mathematics and reading and components of gross motor proficiency, specifically upper limb coordination and speed and agility, along with overall gross motor proficiency scores. There was also evidence that balance was not significantly associated with academic performance in mathematics and reading. However, there was either inconsistent or insufficient evidence to support associations between the other components of gross motor proficiency along with total motor proficiency scores and academic performance. Finally, there was some preliminary evidence from a small number of experimental studies that the implementation of motor skill interventions in the school setting may have a positive impact on academic performance in mathematics and/or reading; however, further research is needed to confirm this possibility. Due to the varying levels of methodological quality found in the studies included in the review, further investigation is warranted, using more robust study designs to explore further the impact of motor skill interventions on academic performance.

### 5.1. Recommendations and Implications for Future Research

To allow more accurate comparisons of findings between studies in the future, researchers should consider consistently using valid and reliable, standardized instruments to assess both fine and gross motor proficiency and academic performance variables. Furthermore, to better understand and explain the underlying mechanisms of the associations between motor proficiency and academic performance, studies should be designed to provide adequate evidence of causality through robust experimental designs that compare the effects of both health-related and performance-related physical fitness (motor proficiency) interventions on the academic performance of school students. Ideally, study designs will also aim to control for known demographic, cognitive, and physical confounders. Future findings from experimental studies may then be able to ascertain whether motor skill training, aerobic fitness, or a combination of both impact cognitive and academic outcomes. Finally, given that students with neurodevelopmental disorders attend mainstream schools, future research should also examine relationships between motor, cognitive, and academic skills in this population to inform potential intervention pathways.

### 5.2. Recommendations and Implications for Policy and Practice

Findings from future high-quality experimental studies aimed at enhancing the PA levels, physical fitness, and academic performance of school students may inform school wellbeing policies and pedagogical approaches to teaching and learning, particularly during the early years of school. This topic is relevant to both education and paediatric health professionals (including physiotherapists and occupational therapists) through their role in the early identification of children experiencing difficulty with motor skills, as this may also impact their academic performance. Furthermore, given that gross motor skills may be linked to academic performance in high school, school policy makers should consider prioritizing, from school entry, students’ acquisition of motor skills.

## Figures and Tables

**Table 1:** Overall levels of evidence from studies examining associations between specific components of motor proficiency and academic performance in mathematics in school-aged children and adolescents.

Motor Proficiency	Associated with Academic Performance in Mathematics (References)	Not Associated with Academic Performance in Mathematics (References)	Summary Coding ^a^	
			*n*/*N* Showing Significant Associations for Outcome (%) ^b^	Association (+,−,0,?) ^c^
Fine motor proficiency				
Fine motor precision	[45,53,56,66]	[55,56]	4/6 (67%)	(+)
Fine motor integration	[48,49,52,53,54,55,56,57,58,60,62,63,64,69,71]	[45]	15/16 (94%)	(++)
Manual dexterity	[43,48,52,55,66,68]	[44,45,62,68]	6/10 (60%)	(+)
Total fine motor score	[21,40,51,55,59,65,67,70,72]		9/9 (100%)	(++)
Gross motor proficiency				
Upper limb coordination	[42,43,44,73]	[42,45]	4/6 (67%)	(+)
Balance	[74]	[44,45,68,74,75]	1/6 (17%)	(0)
Bilateral coordination	[45,69,75]	[45]	3/4 (75%)	(?)
Speed and agility	[42,45,46,68,73,76]	[42,68,75]	6/9 (67%)	(++)
Strength	[45,76]		2/2 (100%)	(?)
Total gross motor score	[42,47,55,57,59,67,68,70,77,78]	[21,41,51,68]	10/14 (71%)	(++)
Total motor proficiency (fine and gross motor scores)	[45,54,61]		3/3 (100%)	(?)

^a^ Summary coding provides an overall summary of findings. ^b^ *n* = number of studies that reported a statistically significant association, *N* = number of studies that reported associations between the specific component of motor proficiency and academic performance in mathematics. ^c^ Association coded as ‘+’ or ‘−‘ indicates a ‘positive or negative association’ (≥60% of reported associations reached statistical significance, based on results of four or more studies); ‘+ +’ or ‘− −‘ indicates ‘strong evidence for a positive or negative association’ (≥60% of reported associations reached statistical significance, based on results of four or more studies with fair-to-good methodological quality); ‘0′ indicates ‘no association’ (0–33% of reported associations reached statistical significance); and ‘?’ indicates ‘inconsistent or uncertain association’ (34–59% of reported associations reached statistical significance or less than four studies examined the relationship).

**Table 2:** Overall level of evidence from studies examining associations between specific components of motor proficiency and academic performance in reading in school-aged children and adolescents.

Motor Proficiency	Associated with Academic Performance in Reading	Not Associated with Academic Performance in Reading	Summary Coding ^a^	
			*n*/*N* Showing Significant Associations for Outcome (%) ^b^	Association (+,−,0,?) ^c^
Fine motor proficiency				
Fine motor precision	[55,66,86]	[53]	3/4 (75%)	(?)
Fine motor integration	[39,48,52,53,54,55,58,60,63,69,71,81,83,85,86,87,88]	[39,57,62,79,85]	17/22 (77%)	(++)
Manual dexterity	[48,52,55,66,68,82,86]	[44,62,68,86,87]	7/12 (58%)	(?)
Total fine motor score	[21,40,55,59,67,86]	[86]	6/7 (86%)	(++)
Gross motor proficiency				
Upper limb coordination	[42,44,73,89]	[42,73]	4/6 (67%)	(++)
Balance	[68,87]	[44,68,75]	2/5 (40%)	(?)
Bilateral coordination	[69,75]		2/2 (100%)	(?)
Speed and agility	[42,68,73,89]	[42,68,75,76]	4/8 (50%)	(?)
Strength	[76]	[76]	1/2 (50%)	(?)
Total gross motor score	[42,55,57,59,67,68,78,84,89]	[21,41,42,47,55,78]	9/15 (60%)	(++)
Total motor proficiency (fine and gross motor scores)	[54,61,80]	[84]	3/4 (75%)	(?)

^a^ Summary coding provides an overall summary of findings. ^b^ *n* = number of studies that reported a statistically significant association, *N* = number of studies that reported associations between the specific component of motor proficiency and academic performance in reading. ^c^ Association coded as ‘+’ or ‘−‘ indicates a ‘positive or negative association’ (≥60% of reported associations reached statistical significance, based on results of four or more studies); ‘+ +’ or ‘− −‘ indicates ‘strong evidence for a positive or negative association’ (≥60% of reported associations reached statistical significance, based on results of four or more studies with fair-to-good methodological quality); ‘0′ indicates ‘no association’ (0–33% of reported associations reached statistical significance); and ‘?’ indicates ‘inconsistent or uncertain association’ (34–59% of reported associations reached statistical significance or less than four studies examined the relationship).

## Supplementary Materials

The following are available online at http://www.mdpi.com/1660-4601/15/8/1603/s1, Table S1: Key data extracted from observational studies examining the relationship between motor proficiency and academic performance in mathematics and reading in school-aged children and adolescents, Table S2: Key data extracted from experimental studies examining the impact of motor proficiency-related interventions on academic performance in mathematics and reading in school-aged children and adolescents.
