# Supplementary material for: Relationships between Motor Proficiency and Academic Performance in Mathematics and Reading in School-Aged Children and Adolescents: A Systematic Review
Source: Int J Environ Res Public Health. 2018 Jul 28;15(8):1603. doi: 10.3390/ijerph15081603 (PMC6121293; doi:10.3390/ijerph15081603)
Supplement: Supplementary file 1 [file ijerph-15-01603-s001.zip › MACDONALD_MILNE_ORR_POPE_Supplementary Table S1_FINAL.docx]

Table S1: Key data extracted from observational studies examining the relationship between motor proficiency and academic performance in mathematics and reading in school-aged children and adolescents

| Authors (Year), Country | Study Design | Study Participants  Sample size (n);  (% girls); Age (Mean ± Standard deviation); SES;  Ethnicity | Outcome Measures | | | Covariates | Main Findings | Critical Appraisal Percentage  (%) |
| --- | --- | --- | --- | --- | --- | --- | --- | --- |
|  |  |  | Motor proficiency | Academic performance (mathematics) | Academic performance (reading) |  |  |  |
| **Aadland et al (b)**  **(2017)**  **Norway** | Longitudinal  (7-months follow-up)  (sample from Active Smarter Kids cluster-randomized controlled trial) | n=1129 children; 48% girls; Age: M=10.2 ± 0.3 years  Schools: n=57 | Motor skills composite:  Catching with one hand; Throwing at a wall target (both from Movement Assessment Battery for Children – 2^nd^ edition); Shuttle run 10 x 5m (from European physical fitness test battery) | Standardized Norwegian National tests (numeracy) | Standardized Norwegian National tests (reading) | ActiGraph accelerometer (physical activity and sedentary time)  Andersen test (aerobic fitness)  Stroop Color and Word Test (executive function (EF) – inhibition)  Verbal Fluency Test (EF - cognitive flexibility)  The Trail Making Test (EF - cognitive flexibility)  Wechsler Intelligence Scale for Children (4^th^ Edition) – Digit Span Test (EF –working memory)  Demographic information (age, body fat, pubertal status, birth weight, SES) | At baseline, significant very weak positive associations between numeracy and aiming (r=0.13, p≤0.05) and catching (r=0.19, p≤0.05); significant association between numeracy and time taken to complete shuttle run (r=-0.28, p≤0.05). At follow up, significant very weak-to-weak positive associations between numeracy and aiming (r=0.18, p≤0.05) and catching (r=0.20, p≤0.05); significant association between numeracy and time taken to complete shuttle run (r=-0.31, p≤0.05)  At baseline, significant very weak positive associations between reading and catching (r=0.10, p≤0.05); significant inverse association between reading and time taken to complete shuttle run (r=-0.19, p≤0.05). At follow up, significant very weak positive associations between reading and aiming (r=0.13, p≤0.05) and catching (r=0.13, p≤0.05); significant association between reading and shuttle run (r=-0.20, p≤0.05)  A modest mediation effect of executive function was found for the relation between the shuttle run and academic performance in numeracy. | 85% |
| **Bellocchi et al**  **(2017)**  **France** | Longitudinal  (16-months follow-up) | n=36 children; Age: (T0) M=64.2±3.9 months; (T1) M=82.2±3.9 months  SES: Sample was not educationally disadvantaged | Developmental Test of Visual Perception (2^nd^ Edition): Visual motor integration (VMI) |  | New Language Examination Battery (phoneme identification task; rhyme task; phonological awareness) (assessed at T0)  Alouette Test-R (reading fluency and accuracy) (assessed at T1) | Developmental Test of Visual Perception (2^nd^ Edition) (general visual perception quotient; motor reduced visual perception) (assessed at T0) | Significant moderate positive associations between VMI (T0) and reading accuracy (T1) (r=0.456, p<0.01)  VMI score (β=0.33, p<0.05) was a significant predictor of reading accuracy  No significant associations between VMI and reading fluency, rhyme and phoneme identification | 50% |
| **Cameron et al**  **(2012)**  **USA** | Longitudinal  (~ 9-months follow-up) | n=213 children; 53% girls; Motor test age (T0): M=4.96±0.42 years; range 3.5-5.75  Achievement test age (T1): M=5.44±0.33 years; range 4.64-6.21 years)  SES: Middle SES background  Ethnicity: 57% Caucasian, 34% Multiracial, 4% Asian, 3% African American, 1% Hispanic, 1% Arabic | Early Screening Inventory-Revised  Fine motor composite (FMC) (replicating a gate with cube blocks; drawing a person; design copy, overall score)  Gross motor composite (GMC) (balance, walk line, hopping, skipping)  (assessed in pre-kindergarten (TO)) | Woodcock Johnson III Test of Achievement (applied problems subtest)  (assessed in fall (T1) and spring (T2) of kindergarten) | Woodcock Johnson III Test of Achievement (letter-word identification; passage comprehension; sound awareness)  (assessed in fall (T1) and spring (T2) of kindergarten) | Parent Questionnaire (maternal education)  Head-Toes-Knees-Shoulders test (executive function)  Woodcock Johnson III Test of Achievement (picture vocabulary) | Significant weak positive associations found for Fine motor composite (FMC) (T0) and reading composite (r=0.35-0.37, p<0.01); letter-word identification (r=0.35-0.37, p<0.01); passage comprehension (r=0.25-0.32, p<0.01); sound awareness (r=0.27-0.29, p<0.01); and applied problems (r= 0.17-0.25, p<0.01) at T1 and T2  Significant very weak-to-weak positive associations found for block task (T0) and all reading outcomes (r=0.15-0.24, p<0.01; applied problems (r=0.11-0.17, p<0.01) at T1 and T2  Significant very weak-to-weak positive associations found for design copy task (T0) and all reading outcomes (r=0.22-0.38, p<0.01); applied problems (r=0.16-0.24, p<0.01) at T1 and T2  Significant very weak-to-weak positive associations found for draw-a-person task (T0) and all reading outcomes (r=0.13-0.20, p<0.01) at T1 and T2  Significant very weak positive associations found for GMC (T0) and reading composite (r=0.17-0.20, p<0.05); passage comprehension (r=0.16, p<0.05); and applied problems (r=0.18-0.19, p<0.05) at T1 and T2  Non-significant associations for GMC (T0) and letter-word identification (T1), sound awareness (T2); and for draw-a-person (T0) and applied problems (T1 and T2)  Findings suggest that executive function and fine motor skills make independent contributions to children’s entry-level achievement as well as improvement from fall to spring of kindergarten. | 70% |
| **Cameron et al**  **(2015)**  **USA** | Longitudinal  (~5-months follow-up)  (sample from a randomized controlled trial) | n=467 children; 50.5% girls; Age (T1): Mean = 4.20 ±0.49 years; range 2.72-4.99  SES: Predominantly low income  Ethnicity: African American (43%), Hispanic (32%); White/ Caucasian (14%), Asian (3%), Multiracial/other (5%), No data (3%)  81% children had English as first language | Beery-Buktenica Developmental Test of Visual-Motor Integration (Short Form) – Visual Motor Integration  (assessed at T1) |  | Test of Preschool Early Literacy: print knowledge; phonological awareness subtests  (assessed at T1 and T2) | Pencil tap test (EF-Inhibitory control)  Wechsler Preschool and Primary Scale of Intelligence-Revised: Backward digit span test (EF-verbal working memory)  Peabody Picture Vocabulary Test-III (receptive vocabulary)  Woodcock Johnson III Psycho-educational Battery: Picture vocabulary subtest (expressive language)  Preschool learning behaviors scale | Significant weak-to-moderate positive associations found for Visual motor integration (VMI) (T1) and phonological awareness (T1 and T2) (r=0.31-0.37, p<0.01); and print knowledge (T1 and T2) (r=0.43-0.44, p<0.01) | 70% |
| **Chang et al**  **(2018)**  **USA** | Longitudinal  (~6-months follow-up) | n=145 children, 49% girls; Age: M=66.23±2.53 months  Schools: n=3  SES: 93% received free lunch (i.e. socially disadvantaged)  Ethnicity: 50.3% Latino/Hispanic, 26.4% Caucasian, 21.4% African American, 1.4% Multiracial | PE Metrics (fundamental movement skills index: object control - dribbling, underhand throwing, locomotor - hopping, sliding)  (assessed at T0) |  | Early Literacy Inventory (global reading proficiency)  (assessed at T1) | Behavior Rating Inventory of Executive Function -Preschool (inhibit, shift, emotional control, working memory, plan/organize)  Demographic information (age, gender, race and language background) | Significant weak positive associations between reading proficiency (T1) and fundamental movement skills (FMS) index (r=0.24, p<0.01), object control (r=0.18, p<0.05) and locomotor  (r=0.23, p<0.01) at T0  FMS assessed at the beginning of kindergarten, especially locomotor skills, accounted for a small but unique amount of variance in reading at the end of kindergarten regardless of age, gender, race and language background  Relationship between FMS and reading proficiency was fully mediated by global executive function | 70% |
| **Dinehart and Manfra**  **(2013)**  **USA** | Longitudinal  (~3-years follow-up)  (sample from Miami-Dade School Readiness Project) | n=3 234 children; 53% girls; Age (T0): Mean=62.5±3.6 months at T1; re-assessed in grade 2 (T1)  SES: Low SES sample  Ethnicity: Hispanic (57%), African American (35%), White/Other (8%) | Learning Accomplishment Profile Diagnostic:  Fine motor manipulation (manual dexterity)  Fine motor writing (grapho-motor abilities / copying)  (assessed at end of pre-kindergarten -T0) | Stanford Achievement Test (SAT10) and GPA (mathematics)  (assessed at T1) | Stanford Achievement Test (SAT10) and GPA (reading)  (assessed at T1) | Learning Accomplishment Profile Diagnostic (expressive language ability;  language comprehension; cognitive counting; cognitive matching; early counting)  Demographics (ethnicity, SES, language spoken at home, days absent from school) | Significant weak positive associations found for fine motor manipulation (FMM) (T0) and SAT10 math (T1) (r=0.22); Unique effect of FMM (T0) (B=1.75, p<0.001) on SAT10 math (T1) with a small effect size (Cohen’s d=0.09)  Significant weak positive associations found for fine motor manipulation (FMM) (T0 and GPA math (T1) (r=0.21); Unique effect of FMM (T0) (B=0.03, p<0.001) on GPA math (T1) with a modest effect size (Cohen’s d=0.14)  Significant very weak positive associations found for FMM (T0) and SAT 10 reading (T1) (r=0.15), and GPA reading (T1) (r=0.15)  Significant weak positive associations found for fine motor writing (FMW) (T0) and SAT10 math (T1) (r=0.33); Unique effect of FMW (T0) (B=1.20, p<0.001) on SAT10 math (T1) with a small effect size (Cohen’s d=0.11)  Significant weak positive associations found for fine motor writing (FMW) (T0) and GPA math (T1) (r=0.31, no p-value); Unique effect of FMW (T0) (B=0.03, p<0.001) on GPA maths (T1) with a modest effect size (Cohen’s d=0.21)  Significant weak positive associations found for FMW (T0) and SAT10 reading (T1) (r=0.30); Unique effect of FMW (T0) (B=0.75, p<0.001) on SAT10 reading (T1) with a modest effect size (Cohen’s d=0.11)  Significant weak positive associations found for FMW (T0) and GPA reading (T1) (r=0.28); Unique effect of FMW (T0) (B=0.03, p<0.001) on GPA reading (T1) with a modest effect size (Cohen’s d=0.11) | 80% |
| **Doyen et al**  **(2017)**  **France** | Longitudinal  (~7-months follow-up) | n=86 kindergarten children; 51% girls; Age (T0): Mean = 6.0 years, range: 5 years, 5 months-6 years, 5 months  Schools: n=3; Classes: n=6  At follow-up: n=73 Year 1 children; 49% girls | Peg-moving task (manual performance – sum of the mean time taken to move 10 pegs over three trials)  (assessed at T0) |  | Phonological awareness (rhyme matching, syllable segmentation, phoneme recognition) (assessed at T0)  Grade 1 assessments: Reading comprehension; word reading; pseudoword reading (assessed at T1) |  | Manual performance (T0) was significantly and inversely associated with phonological awareness (T0) (r=-0.23, p<0.05); reading comprehension (T1) (r=-0.24, p<0.05); word reading (T1) (r=-0.27, p<0.05); pseudoword reading (T1) (r=-0.24, p<0.05)  (i.e. a slower the time on the peg-moving task, the weaker the literacy scores) | 65% |
| **Duran et al**  **(2018)**  **USA** | Longitudinal  (~6.5-months follow-up)  (sample from experimental study) | n=162 children (total); 50% girls  n=89 kindergarten children; 48% girls; Age (T0): M=5.5 years ± 4.0 months;  n=73 Year 1 children; 52% girls; Age (T0): M=6.6 years ±4.5 months  SES: 95% qualified for free or reduced-price lunch – low SES  Ethnicity: 92% African American | Developmental Neuro-psychological Assessment: Design copy subtest (visual motor integration)  (assessed at T0) | Woodcock Johnson III - Test of Achievement (applied problems subtest)  KeyMath3-3 composite (geometry; measurement; numeration)  Test of Early Mathematics Ability (TEMA)  (assessed at T0 and T1) |  | Developmental Neuro-psychological Assessment (attention/EF domain)  Demographics (age, gender, ethnicity, SES, group) | Significant moderate-strong positive associations between visual motor integration (VMI) (T0) and combined mathematics composite (T0 and T1) (r=0.57-0.62, p<0.05).;  Significant moderate-strong positive associations between VMI (T0) and KeyMath composite and individual measures (T0 and T1) (r=0.40-0.61)  Significant moderate positive associations between VMI (T0) and applied problems (T0 and T1) (r=0.50-0.56)  Significant moderate positive associations between VMI (T0) and TEMA (T0 and T1) (r=0.53-0.57)  EF and VMI were robust and unique predictors of improvement in mathematics performance in a sample of low-SES students in kindergarten / grade 1 | 70% |
| **Gandhi et al**  **(2012)**  **Malawi** | Prospective cohort study  (~12-years follow-up)  (sample from the Lungwena Child Survival Study) | n=415 children (51% original sample of 813); 50.6% female; Age (T0): 5 years; Age (T1): 12 years  SES level: 37.1% low, 41% middle, 21.9% high | Developmental Assessment: (gross and fine motor items)  (assessed at T0) | 12-year old assessments  (% of correctly answered maths questions)  (assessed at T1) |  | Developmental Assessment (language and social items)  Demographics (age and height;  birth weight; gender; gestational duration, father’s occupation / literacy, mother’s literacy; wealth index; highest school grade complete; number of times a school grade was repeated) | Fine motor score (T0) was independently associated with mathematics score (T1) (regression coefficient=0.412, p=0.032) (observed data); (regression coefficient=0.445, p=0.011) (imputed data)  Non-significant associations found for gross motor score (T0) and mathematics (T1) (regression coefficient=0.206, p=0.176) (observed data); (regression coefficient=0.184, p=0.216) (imputed data) | 80% |
| **Grissmer et al**  **(2010)**  **USA** | Longitudinal  (~5-years follow-up)  Sample from three data sets:  Early Childhood Longitudinal Study – Kindergarten Cohort (ECLS-K)  National Longitudinal Survey of Youth (NLSY)  British Birth Cohort Study (BCS) | n=7 830 children (ECLS-K study)  n=5 462 children (NLSY study); Age: 22-47 months  n=1 778 (BCS study); Age: 5 years | ECLS-K study: Early Screening Inventory: Gross motor (skipping, hopping, walking backwards, stand on one foot) and Fine motor skills (building blocks, copying figures, draw-a-person)  BCS study:  Fine motor skills (design copy, human figure drawing, profile drawing) | ECLS-K: Achievement tests (mathematical thinking)  Peabody Individual Achievement Test (maths)  BCS: Achievement tests (maths) | ECLS-K: Achievement tests (language and literacy)  Peabody Individual Achievement Test (reading)  BCS: Achievement tests (reading) | Socioemotional  (attention, externalizing I&II, Internalizing, social skills)  ECLS-K: Achievement tests (general knowledge; self-control)  BCS: Achievement tests (school readiness measures - intellectual and behavioral development)  NLSY: Motor and social development instrument assessing children from ages 22-47 months | Significant positive association found for fine motor and reading achievement (β=0.07, p<0.00001); and maths achievement (β=0.14, p<0.00001) in ECLS-K study  Significant positive association found for reading achievement and design copy (β=0.26, p<0.001); human figure drawing (β=0.09, p<0.01) in BCS study  Significant positive association found for maths achievement and design copy (β=0.36, p<0.001); human figure drawing (β=0.09, p<0.01) in BCS study  Gross motor measure was not a significant predictor for mathematics achievement and reading achievement | 40% |
| **Haapala et al**  **(2014)**  **Finland** | Longitudinal  (~3-years follow-up)  (sample from Physical Activity and Nutrition in Children Study and First Steps study) | n=174 children; 43% girls; Age (T0): M=7.7±0.4 years; range 6-8 years  At follow up (grade 3): n=167; 43% girls | 5 x 5m Shuttle run test (speed and agility); flamingo balance test; box and block test (manual dexterity); overall motor performance  (assessed in grade 1-T0) | Basic Arithmetic Test (arithmetic skills)  (assessed in grade 1 (T0), grade 2 (T1) and grade 3 (T2)) | Nationally normed reading battery (reading fluency and comprehension)  (assessed in grade 1 (T0), grade 2 (T1) and grade 3 (T2)) | Physical measures:  Max cycle ergometer (cardiovascular performance); body composition; pubertal status; PA Questionnaire,  Parental education  Risk of reading disability | Overall, poorer motor performance was associated with worse academic skills in children, especially among boys.  Overall motor performance (T0) was associated with reading fluency in grades 1-3 (β=0.28-0.35); reading comprehension in grades 1-3 (β=0.19-0.22); and arithmetic skills in grades 1-3 (β=0.39-0.41)  For boys:  Longer shuttle run time (T0) was associated with poorer reading fluency in grades 1-3 (β=-0.29 to -0.39, p<0.01); reading comprehension in grades 1-2 (β=-0.25 to -0.29, p<0.05); and arithmetic skills in grades 1-3 (β=-0.33 to -0.40, p<0.003);  Poorer balance (T0) was related to poorer reading comprehension in grade 1 (β=-0.20, p=<0.042)  Smaller number of cubes moved in box and block test was related to poorer reading fluency in grades 1-2 (β=0.23 to -0.28, p<0.05); reading comprehension in grade 3 (β=0.23, p=0.037); and arithmetic skills in grades 1-2 (β=0.21-0.23, p<0.043)  For girls:  Longer shuttle run time was associated with poorer reading fluency in grade 3 (β=-0.27, p=0.027); and arithmetic skills in grade 2 (β=-0.25, p=0.0040)  Smaller number of cubes moved in box and block test was related to poorer reading fluency in grades 2 (β=0.26, p=0.030) | 85% |
| **Jaakola et al**  **(2015)**  **Finland** | Longitudinal  (~2-years follow-up) | n=325 high school students; 50% girls; Age (T0): M=13.08±0.25 years  Schools: n=3; classes: n=10 | Fundamental movement skill (FMS) tests (leaping, 10 x 5m shuttle run, dribbling, FMS sum score)  (assessed in grade 7 and 8) | Academic performance (marks in mathematics for grades 7-9) | Academic performance (marks in Finnish language for grades 7-9) | Self-reported PA | Significant very weak-to-moderate associations between Leaping test (grades 7 and 8) and marks in maths (grades 7-9) for girls (r=0.18-0.41, p<0.05); and weak associations between leaping test (grade 8) and marks in maths (grades 7 and 9) for boys (r=0.24-0.30)  Significant very weak positive associations between dribbling task (grade 7) and marks in Finnish language (grade 7) for girls (r=0.17, p<0.05); and marks in maths (grade 9) for boys (r=0.18, p<0.05)  Significant very weak positive associations between shuttle run (grade 7) and marks in Finnish language (grade 7) for boys (r=0.17, p<0.05); and marks in maths (grade 9) for boys (r=0.16, p<0.05)  Significant very weak positive associations between shuttle run (grade 8) and marks in maths (grade 8 and 9) for boys (r=0.18-0.19, p<0.05); and marks in maths (grade 9) for girls (r=0.20, p<0.05); and Finnish language (grade 7) for boys (r=0.17, p<0.05)  Significant very weak positive associations between FMS sum score (grade 7) and maths (grade 7 and 9) for boys (r=0.16-0.18, p<0.05); maths (grade 8 and 9) for girls (r=0.16-0.19, p<0.05)  Significant very weak-to-weak positive associations between FMS sum score (grade 8) and maths (grades 7-9) for boys (r=0.18-0.25, p<0.01); maths (grades 8 and 9) for girls (r=0.23-0.25, p<0.05) | 80% |
| **Kim et al**  **(2017)**  **USA** | Longitudinal  (2-years follow-up)  (sample from three experimental studies) | n=135 kindergarten students; 50% girls; Age: Mean=5.6±0.37 years; range 5.0-6.8 years  n=119 grade 1 students; 46% girls; Age: M=6.7±0.43 years; range 6.0-7.9 years  SES: 71% eligible for a lunch subsidy  Ethnicity: 71% African American, 26% Caucasian, 3% other (Hispanic or Multiracial) | Neuro-psychological assessment battery: Visual motor integration (design copy);  Visual motor precision (fine motor coordination)  (assessed at beginning of kindergarten (T0), end of kindergarten (T1) and end of grade 1 (T2)) | Key Math-3 Diagnostic Assessment (numeration; geometry; measurement)  (assessed at beginning of kindergarten (T0), end of kindergarten (T1) and end of grade 1 (T2)) |  | Demographics (study site;  lunch subsidy status; treatment group status)  Neuro-psychological assessment battery (visual attention -attention/ EF) | Visual motor integration (VMI) and maths were positively and reciprocally related. Fine motor coordination (FMC) at beginning of kindergarten indirectly contributed to mathematics at the end of year 1 through its effect on VMI at the end of kindergarten  Partial correlations (controlling for age):  Significant moderate positive associations for kindergarten students between VMI (T0-T2) and maths (T0-T2) (r=0.417-0.575, p<0.01)  Significant weak positive associations for kindergarten students between FMC (T0-T2) and maths (T0-T2) (r=0.250-0.383, p<0.01)  Significant moderate-to-strong positive associations for year 1 students between VMI (T0-T2) and maths (T0-T2) (r=0.529-0.669, p<0.01),  Significant weak positive associations between FMC (T0-T2) and math (T0) (r=0.208-0.237, p<0.05)  Non-significant associations for kindergarten students between FMC (T0, T2) and maths (T2); and for year 1 students between FMC (T1) and maths (T0-T2)  For both cohorts, FMC did not directly predict mathematic skills | 80% |
| **Kurdek & Sinclair**  **(2001)**  **USA** | Longitudinal  (~4-5-years follow-up) | n=281 children; 53% girls. Baseline data collected in kindergarten (T0)  At follow-up in grade 4 (T1): Age: M=11.22±0.35 years; range 10.48-12.05 years  Middle-class SES  93% White | Kindergarten Diagnostic Instrument:  Visual motor integration (VMI);  Gross motor skills (jumping, skipping, hopping)  (assessed at T0) | Ohio proficiency-based assessments in mathematics  (assessed at T1) | Ohio proficiency-based assessments in reading  (assessed at T1) | Kindergarten Diagnostic Instrument:  Verbal skills (auditory memory; concept mastery; form perception; general information; number skills; verbal association; verbal opposite; vocabulary)  Visual motor skills (body awareness, visual discrimination, visual memory) | Significant weak positive association between visual motor integration (T0) and maths (T1) (r=0.21, p<0.01).  Significant weak positive associations between gross motor skills (T0) and reading (T1) (r=0.17, p<0.01) and maths (T1) (r=0.17, p<0.01)  Non-significant associations between VMI (T0) and reading (T1) | 75% |
| **Lachance et al**  **(2006)**  **USA** | Longitudinal  (~3-years follow-up) | At baseline (Kindergarten):  n=249 children; 52% girls; Age (T0): M=5.83±0.35 years (boys); M=5.72±0.33 years (girls)  At follow up (grade 3): n=214 children; 51% girls; Age: M=8.73±0.30 years (boys); M=8.61±0.32 years (girls)  Ethnicity: 89% Caucasian (boys), 83% Caucasian (girls) | Beery-Buktenica Developmental Test of Visual-Motor Integration (4th Ed)  (assessed in kindergarten (T0), grade 1 (T1), grade 2 (T2) and grade 3 (T3)) | Test of Early Mathematical Ability-2nd Edition (TEMA-2)  Keymath revised (numeration, geometry; addition; subtraction; measurement; time and money)  Woodcock Johnson-revised (math calculation)  Counting trials  Maths facts | Woodcock Johnson - Revised (letter word identification; word attack)  Reading fluency  Rapid automatized reading (single word retrieval fluency) | Wechsler Abbreviated Scale of Intelligence (overall cognitive ability)  Developmental Test of Visual Perception (2nd edition) - motor reduced subtests (perceptual skills) | Positive associations between visual motor integration (VMI) (T0-T3) and TEMA-2 (T0-T3) (r=0.29-0.49, p=0.05)  Positive associations between VMI (T0-T3) and Letter word identification (T0-T3) (r=0.28-0.53, p=0.05)  Positive associations between VMI (T0-T3) and word attack (T0-T3) (r=0.21-0.38, p=0.05) | 65% |
| **Luo et al**  **(2007)**  **USA** | Longitudinal  (~18-months follow-up)  (sample from  Early Childhood Longitudinal Study – Kindergarten Class (ECLS-K)) | n=9 816 European American (EUA) kindergarten children; 49% girls; Age: (T)): M=68.61 months (EUA)  n=244 East Asian American (EAA) children; 51% girls; Age: M=67.07 months (EAA)  Ethnicity: White / non-Hispanic (EUA); Asian (EAA) | Early Screening Inventory-Revised (ESI-R) – Fine motor skills (replicating a gate with cube blocks, drawing a person, copying 5 simple figures; composite score)  (assessed at beginning of kindergarten) | Child Assessment Battery (mathematics achievement)  (assessed in kindergarten and grade 1) |  | Demographics (SES, parental education level; parental educational expectations for child) | Fine motor skills were positively related to mathematics at kindergarten entry, with the strength of the relationship similar between EAA and EUA children.  Fine motor skills were predictive of mathematics performance over time | 80% |
| **Magistro et al**  **(2015)**  **Italy** | Longitudinal  (~8-months follow-up) | n=63 children; 48% girls; Age: M=8.4±0.4 years  Schools: n=3  Ethnicity: 83% Italian, 9% Romanian, 8% Albanian | Test of Gross Motor Development (2^nd^ edition) (gross motor skills)  (assessed at beginning of school year – T0) | End of school year teacher questionnaire – (scholastic achievement in mathematics)  (assessed at end of school year – T1) |  | Family structure  End of school year self-report questionnaire (Attention deficit hyperactivity disorder behavior, scholastic achievements – overall, PE) | Significant moderate positive associations between gross motor skills and maths achievement (r=0.41, p<0.01) | 40% |
| **Manfra et al**  **(2017)**  **USA** | Longitudinal  (~3-4 years follow-up)  (sample from the Miami School Readiness Project) | n=1442; 52.3% girls; Age (T0): 4 years  SES: 88.2% registered for free lunch program (low income)  Ethnicity: 63.2% Hispanic/Latino, 32.3% Black, 3.3% White, 1.2% Other  T1: Preschool  T2: Year 3 | Learning Accomplishment Profile Diagnostic (LAP-D):  Fine motor subtest (fine motor manipulation; fine motor copying)  (assessed in pre-kindergarten T0) | FCAT (student achievement -maths domains)  Stanford Achievement Test (10th Ed) (SAT-10)  Grade 3 classroom performance (GPA)  * Grades (0=F, 4=A)  (assessed in grade 3 – T1) | FCAT (student achievement - reading domains)  Stanford Achievement Test (10th Ed) (SAT-10)  Grade 3 classroom performance (GPA)  * Grades (0=F, 4=A)  (assessed in grade 3 – T1) | Demographic information (age, gender, immigration, race/ethnicity, number of days absent, grade 3 free/reduced lunch status, parental income)  LAP-D - cognitive (matching / counting subtest); Language (comprehension, naming) | Significant associations between fine motor manipulation (FMM) and SAT-10 reading (r=0.18, p<0.001), reading GPA (r=0.18, p<0.001), SAT-10 maths (r=0.24, p<0.001), maths GPA (r=0.24, p<0.001)  Significant associations between fine motor copying (FMC) and SAT-10 reading (r=0.33, p<0.001), reading GPA (r=0.26, p<0.001), SAT-10 maths (r=0.35, p<0.001), maths GPA (r=0.27, p<0.001),  While controlling for demographic factors, days absent and school, FMC was a significant predictor of Grade 3 SAT-10 reading (β=0.74, p<0.001) and Grade 3 SAT-10 mathematics (β=0.72, p<0.001)  FMM was a significant predictor of Grade SAT-10 mathematics (β=0.48, p<0.001) | 65% |
| **Pagani et al (a)**  **(2010)**  **Canada** | Longitudinal  (2-year follow-up)  (sample from the Quebec Longitudinal Study of Child Development) | N=1 145 children; 47% girls; Age: 65 months | Teacher-rated motor assessment:  Gross motor skills (well-coordinated; climbs stairs; overall physical development)  Fine motor skills (proficiency at holding a pen; ability to manipulate object)  (assessed in kindergarten – T0) | Number Knowledge Test (NKT) - (maths)  (assessed in kindergarten – T0)  Teacher-reported academic achievement (maths)  (assessed in grade 2 – T1) | Teacher-reported achievement (reading)  (assessed in grade 2 – T1) | Family characteristics (maternal education, SES, income)  Peabody Picture Vocabulary Test (vocabulary knowledge)  Social Behavior Questionnaire (children’s behavioral adjustment)  Teacher-rated scale on classroom engagement | Significant very weak-to-weak positive association found between NKT (T0) and fine motor (T0) (r=0.30, p<0.0001); and gross motor (T0) (r=0.19, p<0.0001)  Significant very weak-to-weak positive association found between teacher-rated maths marks (T1) and fine motor (T0) (r=0.34, p<0.0001); and gross motor (T0) (r=0.20, p<0.0001)  Significant very weak-to-weak positive association found between teacher-rated reading marks (T1) and fine motor (T0) (r=0.35, p<0.0001); and gross motor (T0) (r=0.23, p<0.0001)  Fine motor skills in kindergarten were predictive of grade 2 achievement, reading (β=0.11, p<0.01) and maths (β=0.17, p<0.001) | 90% |
| **Papadimitiriou et al**  **(2014)**  **Greece** | Longitudinal  (2-years follow-up) | Kindergarten (T0):  n=300 children; 49% girls; Age: M=5.6±0.36 years; range 5.1-6.7 years  Grade 1 (T1):  n=288 children; 51% girls; Age: M= 6.7±0.37 years; range 6.1-7.7 years  Grade 2 (T2)  n=287 children (49% girls); Age: M=7.6±0.37 years; range 7.1-8.2 years | Motor skills test (bead threading task; shape copying task; postural stability task – inclination from upright)  (assessed at T0) |  | Phonological Awareness test (syllable segmentation; recognition of common initial phoneme; deletion of syllable and deletion of phoneme)  (assessed at T0)  Standardized Greek reading tests: word reading accuracy / fluency; pseudoword reading accuracy / fluency; reading comprehension)  (assessed at T1 and T2) | Rapid naming test  Receptive vocabulary task (oral language skills)  Expressive vocabulary task (oral language skills)  Digit span task (phonological short term memory)  Pseudo-words repetition task (phonological short term memory)  Sound-order test (auditory discrimination) | Significant weak positive associations between fine motor skills (shape copying) (T0) and phonological awareness (T0) (r=0.337, p<0.01), grade 1 reading performance (T1) (r=0.245, p<0.01), grade 2 reading performance (T2) (r=0.232, p<0.01)  Significant associations between the inclination from upright on a postural stability task and phonological awareness (r=-0.251, p<0.01), grade 1 reading performance (r=-0.144, p<0.05), grade 2 reading performance (r=-0.0117. p<0.05)  (i.e. the larger the inclination from upright on the postural stability task, the lower the scores on reading tasks)  Non-significant findings between fine motor skills (bead threading) and phonological awareness; reading performance in grades 1 and 2  Shape copying skills in kindergarten predictive of reading accuracy in grade 2 (β=0.19, p<0.01) | 45% |
| **Roebers et al**  **(2014)**  **Switzerland** | Longitudinal  (2-years follow-up) | Pre-kindergarten (T0): n=169 children; 45.6% girls); Age: M=69.4±4.28 months  Grade 1 (T2): n=116 children; Age: M=7 years, 9 months  Ethnicity: >97% sample white | Movement Assessment Battery for Children – 2^nd^ edition (manual dexterity scale)  (assessed at T0 and T1) | Heidelberger Rechentest – Standardized mathematics test  (assessed at T2) | Wurburger Leise Lese Probe – Standardized reading test  Salzburger Lese-Screening – Standardized reading test  (assessed at T2) | Cognitive Flexibility Task (Executive function (EF))  Fruit-Stroop Task (EF)  Backwards Color Recall task (EF)  Culture-Fair Intelligence Test (intelligence)  Test of Non-Verbal Intelligence (intelligence) | Significant weak positive associations between reading achievement (T2) and threading beads (T0) (r=0.36, p<0.001); posting coins (T0) (r=0.27, p<0.001); threading lace (T1) (r=0.34, p<0.001); placing pegs (r=0.35, p<0.001); drawing trail (T0 and T1) (r=0.28-0.35, p<0.001)  Significant weak positive associations between maths achievement (T2) and threading beads (T0) (r=0.28, p<0.001), posting coins (T0) (r=0.24, p<0.01), threading lace (T1) (r=0.37, p<0.001), placing pegs (T1) (r=0.35, p<0.001); drawing trail (T0 and T1) (r=0.18-0.23, p<0.05) | 85% |
| **Schatschneider et al**  **(2004)**  **USA** | Longitudinal  (3-years follow-up)  (sample from larger longitudinal study) | n=540 children (total)  n=384 children in kindergarten (T0) to grade 1 (T1) cohort; 50% girls; Ethnicity: 54.4% Caucasian, 16.8% African American, 15,2% Hispanic, 12.4% Asian, 1.3% Other  n=189 children in kindergarten (T0), grade 1 (T1) and grade 2 (T2) cohort; 48% girls; Ethnicity: 54% Caucasian, 14.3% African American, 16.4% Hispanic, 14.3% Asian, 1% other | Beery Test of Visual Motor Integration (visual-motor integration) |  | Phonological awareness  Alphabetic knowledge (letter names, letter sounds)  (assessed at T0)  Woodcock-Johnson Psycho-Educational Battery – Revised: (letter word identification, passage comprehension)  Test of Word Reading Efficiency (word reading efficiency)  (assessed at T1 and T2) | Clinical Evaluation of Language Functions - Revised (expressive syntax; syntactic comprehension)  Rapid Automatized Naming  Peabody Picture Vocabulary Test - Revised (oral vocabulary)  Recognition-discrimination test (visual perceptual task) | Very weak-to-weak correlations found between visual motor integration (VMI) and reading variables (letter sounds/names, phonological awareness, passage comprehension, word identification, word reading efficiency) from October Kindergarten to end of Grade 1/ 2 (r=0.27-0.37) as well as April Kindergarten to end of Grade 1 / 2 (r=0.13-0.34)  VMI consistently less related to early reading achievement than phonological awareness, rapid automatized naming letter and knowledge of letter names and sounds  When controlling for phonological awareness, VMI not a significant predictor for reading outcomes in Grade 1 & 2 | 65% |
| **Sigmundsson et al**  **(2017)**  **Norway** | Longitudinal  (~2 ½ years follow-up) | (T0): n=67; 46% girls  Age: M=9.7±0.3 years; range: 9.3-10.2 years  (T1): n=58; 48% girls  Age: M=12.1±0.2 years | TPF - physical fitness (jumping, throwing, climbing, running)  Movement Assessment Battery for Children (manual dexterity, ball skills, balance)  (assessed at T0 and T1) |  | Word Chain Test (reading achievement)  (assessed at T0 and T2) |  | Significant moderate positive association found between reading and physical fitness in 9-year old girls (r=0.404, p<0.05)  All other associations between overall motor competence (MABC) and physical fitness (TPF) and reading in children who are 9 and 12 years of age were not significant | 55% |
| **Son and Meisels**  **(2006)**  **USA** | Longitudinal  (~18-months follow-up)  (sample from the Early Childhood Longitudinal Study-Kindergarten) | Kindergarten (T0)  n=12 583 children; 49.6% girls; Age: M=65±4.07 months; range 49 to 83 months    Grade 1 (T1)  SES: 20% income below poverty line  Ethnicity: 15.5% African American, 17.5% Hispanic | Early Screening Inventory-Revised:  Visual motor skills (building a gate, draw-a-person, copying figures)  Gross motor skills (balancing, hopping, skipping, walking backwards)  (assessed at T0) | National Center for Education Statistics (mathematics assessments)  (assessed at T0 and T1) | National Center for Education Statistics (reading assessments)  (assessed at T0 and T1) | Demographics (age; gender; ethnicity; home language; SES) | Significant weak-to-moderate positive associations between visual motor skills (T0) and reading (T0 and T1) (r=0.35-0.40, p<0.001); and maths achievement (T0 and T1) (r=0.44-0.48, p<0.001)  Significant weak positive associations between gross motor skills (T0) and reading (T0 and T1) (r=0.15-0.19, p<0.001); and maths (T0 and T1) (r=0.20-0.22, p<0.001) | 90% |
| **Verdine et al**  **(2014)**  **USA** | Longitudinal | Pre-kindergarten (T0): n=44 children; 50% girls; Age: M=45.5±2.37 months; range 38-48 months;  Age (T1): M=57.1±2.54 months; range 52-62 months  SES: 45% low SES | Beery Test of Visual-Motor Integration – (visual-motor integration)  (assessed at T1) | Wechsler Individual Achievement Test (3^rd^ Edition) (math problem solving subtest)  (assessed at T1) |  | Demographics (SES, gender)  Peabody Picture Vocabulary Test (vocabulary)  Flexible Item Selection Task (cognitive flexibility)  The Tap Test (inhibitory control)  Test of Spatial Assembly (early geometric and spatial reasoning) | Significant strong positive association between visual motor integration (VMI) and maths (r=0.673, p<0.01)  Significant partial correlations (controlling for SES, gender and vocabulary) between VMI and maths (r=0.43, p=0.005)  VMI was a significant predictor of maths (β=0.346, p=0.05) | 80% |
| **Wang et al**  **(2015)**  **China** | Longitudinal  (1-year follow-up) | Kindergarten (T0)  n=85 children; 53% girls; Age: M= 5 years 2 months; range 4 years 9 months-6 years 2 months    (T1): n=73 children  SES: Middle income families | Visual-motor skill (Copying Korean and Hebrew words)  (assessed at T0) |  | Chinese word recognition task (word reading)  (assessed at T0 and T1)  Phonological Awareness  (assessed at T0) | Mother’s highest level of education  Visual-orthographic copying skill – unfamiliar Chinese    Rapid Automatized Naming for numbers  Raven's Standard Progressive Matrices (non-verbal IQ)  Chinese word writing  Semantic radical awareness  Stanford-Binet Intelligence Scale - vocabulary subtest (expressive vocabulary)  Morphological awareness | After controlling for age and IQ, no significant associations between Chinese reading and copying skills (r=0.15-0.23, ns) | 75% |
| **Aadland et al (a)**  **(2017)**  **Norway** | Cross-sectional  (61.7% sample from Active Smarter Kids cluster-randomized controlled trial) | n=697 children; 51% girls; Age: M=10.2±0.3 years  Schools: n=57 | Motor skills composite: Catching with one hand; Throwing at a wall target (both from Movement Assessment Battery for Children – 2^nd^ edition); Shuttle run 10 x 5m (from European physical fitness test battery) | Standardized Norwegian National tests (numeracy) | Standardized Norwegian National tests (reading) | ActiGraph accelerometer (physical activity and sedentary time)  Andersen test (aerobic fitness)  Stroop Color and Word Test (executive function (EF) – inhibition)  Verbal Fluency Test (EF - cognitive flexibility)  The Trail making test (EF- cognitive flexibility)  Wechsler Intelligence Scale for Children (4^th^ Edition) - Digit Span Test (EF–working memory)  Demographic information (age, body fat, pubertal status, birth weight, SES) | Significant positive associations between motor skills composite and numeracy for boys (standardized regression coefficient β=0.17, p<0.05); and girls (β=0.22, p<0.05)  Significant positive associations between motor skills composite and reading for girls (β=0.14, p<0.05)  Non-significant associations between motor skills composite and reading for boys | 70% |
| **Becker et al**  **(2014)**  **USA** | Cross-sectional | n=127 pre-kindergarten and kindergarten children; 46% girls; Age M=68.55±7.75 months; range 53-80 months  n=49 prekindergarten children; 45% girls; Age: M= 59.88 months  n=78 kindergarten children; 47% girls; Age: M=74 months  SES: Middle and low income households  Ethnicity: 67% white, 2% African American, 15% Latino/Hispanic, 5% Asian/Pacific Islander, 11% other | Beery Visual-Motor Integration (6^th^ Edition) Visual-motor skills (VMS) | Woodcock Johnson Psycho-Educational Battery-III Tests of Achievement) (applied problems subtest) | Woodcock Johnson Psycho-Educational Battery-III Tests of Achievement (letter-word identification subtest) | Maternal education, enrolment in Head Start, English language  Woodcock Johnson Psycho-Educational Battery-III Tests of Achievement – (picture vocabulary subtest - expressive and receptive vocabulary)  Head-Toes-Knees-Shoulders task (behavioral self-regulation)  Day-Night Stroop task (inhibitory control)  Woodcock-Johnson Auditory Working Memory (working memory) | Significant strong positive association between VMS and letter-word identification (r=0.62, p<0.05); and mathematics (r=0.59, p<0.05)  Children’s VMS scores were positively associated with children’s emergent literacy scores (β=0.18, p=0.015), (adjusting for English language learner status, gender, age and maternal education)  Children’s VMS scores were significantly related to maths (β=0.13, p=0.045), (adjusting for English language learner status, gender, age and maternal education) | 60% |
| **Cadoret et al**  **(2018)**  **Canada** | Cross-sectional  (sample from longitudinal research project - Young Children and their Environments) | n=152 children; 55% girls; Age: 7 years | Bruininks Oseretsky Test of Motor Proficiency (2^nd^ Edition) (Short Form) (Total motor proficiency standard score) | Wechsler Individual Achievement Test (2^nd^ Edition) (mathematics composite) | Wechsler Individual Achievement Test (2^nd^ Edition) (reading composite) | Wechsler Intelligence Scale for Children (4^th^ Edition) (cognitive ability) | Significant weak positive association between Total motor proficiency and reading composite  (r=0.28, p<0.01)  Significant weak positive association between Total motor proficiency and mathematics composite (r=0.21, p<0.05)  The relation between motor proficiency and academic performance is mediated by cognitive ability (specifically working memory and perceptual reasoning processes) | 60% |
| **Chagas et al**  **(2016)**  **Brazil** | Cross-sectional | n=122 students; 57.4% girls; Age: range 12-14 years M=13.8±0.7 years (girls); M=13.8±0.6 years (boys) | Korper-koordination Test fur Kinder (Gross motor coordination – walking backwards, one-leg hop, two-leg jump, moving sideways) | Portuguese Mathematics standardized regional test (academic achievement in maths) | Portuguese Language standardized regional test (academic achievement in reading) | Body mass (kg)  Physical Activity Questionnaire for Older Children (PA levels) | No significant associations found for gross motor coordination and reading/ mathematics | 50% |
| **Dunn et al**  **(2006)**  **South Africa** | Cross-sectional | n=238 children; 47% girls Age: M=5.8±0.3years; range 4 years 9 months-7 years, 0 months    SES: 86 participants from Upper, 58 middle, 25 lower  Ethnicity: 28% White, 30% Black, 42% coloured | Beery Developmental Test of Visual-Motor Integration (3^rd^ Edition): Visual motor integration  Copying test (visual motor integration) | Teacher's ratings on academic abilities  (arithmetic skills - 7 point Likert scale) | Teacher's ratings on academic abilities (reading skills - 7 point Likert scale) | Teacher's ratings on academic abilities (school readiness, fine motor skills, concentration, writing) | Significant moderate positive associations found for visual motor integration (VMI) and arithmetic (r=0.44, p<0.01); reading (r=0.42, p<0.01)  Significant moderate-to-strong positive associations between Copying test and arithmetic (r=0.58, p<0.01); and reading (r=0.60, p<0.01) | 40% |
| **Esteban-Cornejo et al**  **(2014)**  **Spain** | Cross-sectional  (sample from the UP & DOWN longitudinal study) | n=2038 children and adolescents; 48.5% girls; Age: Mean 10.20±3.31 years; range 6-18 years | Assessing Levels of Physical Activity (ALPHA) fitness test battery - Motor ability (4 x 10m shuttle run test of speed of movement, agility and coordination) | Individual grades (mathematics) |  | Individual grades (language) and combined grade point average  ALPHA fitness test battery (muscular strength, cardiorespiratory capacity)  Demographics (SES, pubertal status, BMI,  waist circumference) | Significant positive associations found for motor ability and mathematics (standardised regression coefficient β=0.254, p<0.001) (controlling for gender, age, city, pubertal status and maternal education)  Motor ability was independently and significantly associated with mathematics (β=0.185, p<0.001) (controlling for gender, age, city, pubertal status and maternal education, cardiorespiratory capacity, muscular strength) | 60% |
| **Geerstsen et al**  **(2016)**  **Denmark** | Cross-sectional | n=423 children; 49.4% girls; Age: Mean= 9.29±0.35 years; range 8-10 years  Schools: n=7; Classes: n=20 | Visuomotor accuracy-tracking task (fine motor skills)  Coordination wall task (gross motor skills) | Danish Standardized test of academic performance (mathematics) | Danish Standardized test of academic performance (reading comprehension) | Anthropometric measures (body mass, BMI, tanner stage)  YoYo intermittent recovery level 1 children’s test (exercise capacity)  Cambridge Neuro-psychological Test Automated Battery (reaction time, sustained attention ability, spatial working memory, paired associated learning, free-recall word memory) | Fine motor skill was associated with better academic performance in mathematics (estimated slope coefficient 0.20±0.03, p<0.001) and reading comprehension (estimated slope coefficient of 0.26±0.05, p<0.001)  Better performance in gross motor skills (i.e. shorter time to complete the wall) was associated with better scores in mathematics (estimated slope coefficient -0.22±0.03, p<0.001) and reading comprehension (estimated slope coefficient -0.32±0.05, p<0.001) | 55% |
| **Lonneman et al**  **(2011)**  **Germany** | Cross-sectional | n=53 children; 40% girls; Age: M=9.0 years; range 8 to 10 years | Static balance tasks (standing on right or left leg, eyes open / closed) | DEMAT 2+ German scholastic achievement test for mathematics  Arithmetic tasks (addition with/without carrying, subtraction with/without borrowing, multiplication) |  | D2 Test of Attention (attentional capabilities)  Raven’s Standard Progressive Matrices Plus (reasoning capabilities) | Balancing with eyes open was related to DEMAT 2+ score (r=0.25, p<0.05), multiplication (r=0.25-0.31, p<0.05) and addition/subtraction tasks without carrying/borrowing (r=0.23-0.24, p<0.05)  Balancing with eyes closed was related to multiplication (r=0.36-0.37, p<0.05) and subtraction with borrowing (r=0.26-0.34, p<0.05)  Significant partial correlations between balance tasks with eyes closed and multiplication (r=0.30-0.34, p<0.05); subtraction with borrowing (r=0.26-0.27, p<0.05) but not for less complex addition/subtraction tasks (controlling for age, attentional and reasoning capabilities) | 55% |
| **Mayes et al**  **(2009)**  **USA** | Cross-sectional  (sample from a general population epidemiologic study of sleep disorders in children) | n=214 children; 53% girls; Age: M=8.6±1.5 years; range 6-12 years  Ethnicity: 78% White, 17% Black, 5% Asian | Beery Developmental Test of Visual-Motor Integration (4^th^ Edition): Visual-motor integration (VMI)  Grooved Pegboard Test (fine motor ability) | Wide Range Achievement Test (3^rd^ Edition) (arithmetic subtest) | Wide Range Achievement Test (3^rd^ Edition) (reading subtest) | Gordan Diagnostic System (attention)    Wechsler Intelligence Scale for Children-III Digit span (working memory, attention); Symbol search (processing speed); Coding (grapho-motor speed)  California Verbal Learning Test (memory)  Wisconsin Card Sorting Test-64 (set shifting and mental flexibility)    Stroop Color and Word Test (response inhibition)  Animal Naming Test (verbal fluency)  Pediatric Behavior Scale (ADHD Subscale)  Wechsler Abbreviated Scale of Intelligence (IQ) | IQ, VMI and Coding significant predictors of maths  VMI and fine motor skills not predictors of reading achievement scores | 60% |
| **McPhillips and Jordan-Black**  **(2007)**  **Ireland** | Cross-sectional | n=239 year 1 children; 39% girls; Age: M=57.4±3.6 months  n=276 year 4 children; 50% girls; Age: M=101.4±4.5 months | Movement Assessment Battery for Children (manual dexterity, ball skills,  balance skills) |  | Wechsler Objective Reading Dimensions (basic reading subtest)  (assessed only in year 4 children) | Shilder Test (persistence of asymmetrical tonic neck reflex)  British Picture Vocabulary Scale (receptive language)  Brown attention deficit disorder scale  SES | Simple regression analyses found that motor skills may be weakly predictive of attainment of reading without confounds  Multiple regression analyses found that motor skills are not predictive of reading in context of other predictors | 75% |
| **Memis et al**  **(2016)**  **Turkey** | Cross-sectional | n=168 children in grade 1; 50% girls | Developmental Visual Perception Test (2^nd^ Edition) – Visual motor integration |  | Informal Reading Inventory (reading levels, reading comprehension, reading errors) | Developmental Visual Perception Test (2^nd^ Edition) (general visual perception, motor-reduced visual perception) | Significant moderate positive associations between visual motor integration (VMI) and reading speed (r=0.454, p<0.01), reading comprehension (r=0.469, p<0.01)  Significant association between VMI and reading errors (r=-0.418, p<0.01) | 20% |
| **Morales et al**  **(2011)**  **Spain** | Cross-sectional | n=487 children and adolescents; 51% girls  n=243 children 9-12 years)  n=244 children 13-16 years  Schools: n=6  SES: Low to Medium | Tower of cubes test (fine motor skills – time taken to build a tower out of cubes)  Target Throwing test (gross motor skills – time taken to throw ball at target and catch three times) | Battery of Differential and General Skills (maths skills test) |  | Clinical history questionnaire  Extra-curricular physical activity (modified from International Physical Activity Questionnaire)  Battery of Differential and General Skills (oral skills test) | For cohort aged 9-12 years:  Maths performance was significantly and inversely associated with the time taken to build a tower out of cubes (r=-0.727, p<0.05). Maths performance was significantly and inversely associated with the time taken to throw and catch a ball three times (r=-0.439, p<0.05)  For cohort aged 13-16 years:  Maths performance was significantly and inversely associated with the time taken to build a tower out of cubes r=-0. (643, p<0.05). Maths performance was significantly and inversely associated with the time taken to throw and catch a ball three times (r=-0.163, p<0.05)  (i.e. the slower the time taken to complete fine and gross motor tasks, the lower the score on the maths skills test) | 50% |
| **Murrihy et al**  **(2017)**  **Australia** | Cross-sectional | n=133 children; 54.1% girls; Age: M=9.7 years, range 8-12.5 years | McCarron Assessment of Neuromuscular Development: Psychomotor ability (finger-nose-finger, jumping, heel toe walking, standing on one foot) | Australian Woodcock Johnson III Tests of Achievement Battery: Mathematics achievement (calculation, applied problems) | Australian Woodcock Johnson III Tests of Achievement Battery: Reading achievement (letter-word identification, passage comprehension) | Automated Working Memory Assessment (short-term memory)  Australian Woodcock Johnson III tests of Cognitive Abilities (general intellectual ability, working memory, crystallized intelligence, fluid intelligence) | Significant weak positive associations between letter word identification and finger-nose test (r=0.33, p<0.001); and heel-toe walking (r=0.32, p<0.001)  Significant weak positive associations between finger-nose test and calculation (r=0.26, p<0.05)  Psychomotor ability (finger nose, walking, jumping, balance) did not have a positive direct effect on reading achievement or maths achievement.  However, the size of standardized indirect effect of psychomotor ability on reading (β=0.25, p<0.05) and maths β=0.22, p<0.05) was found to be statistically significant, supporting the prediction that psychomotor ability leads to higher reading achievement through the mechanism of short-term memory | 55% |
| **Pagani and Messier**  **(2012)**  **Canada** | Cross-sectional  (sample from Montreal Longitudinal Preschool Study) | N=522 kindergarten children | Teacher-rated motor assessment:  Gross motor skills (well-coordinated; climbs stairs; overall physical development)  Fine motor skills (proficiency at holding a pen; ability to manipulate object)  Perceptual motor skills (copying, writing) | Number Knowledge Test (maths)  Teacher-reported academic achievement (maths) | Teacher-reported academic achievement (reading) | Peabody Picture Vocabulary Test (verbal competence)  Social Behavior Questionnaire (behavioral adjustment)  Control variables (family/child characteristics) | Significant weak positive association between early maths skills and teacher-rated fine motor skills (r=0.29, p<0.001), perceptual motor abilities (r=0.31, p<0.001) and gross motor ability (r=0.20, p<0.001)  After controlling for other confounding factors, significant associations were found between maths skills and fine motor (β=0.16, p<0.01) and perceptual motor (β=0.14, p<0.01) | 60% |
| **Pienaar et al**  **(2013)**  **South Africa** | Cross-sectional  (sample from North West-CHILD longitudinal study) | n=812 year 1 children; 48.5% girls; Age: M=6.78±0.49 years  SES: n=19.1% quintile 1 (poorest); n=21.2% quintile 5 (highest)  Ethnicity: 70% Black, 27% White, 2% Coloured, 1% Indian | Bruininks Oseretsky Test of Motor Proficiency (2^nd^ Edition) - Short Form  Beery-Buktenica Developmental Test of Visual-Motor Integration (4^th^ Edition) (visual-motor integration (VMI) total score, visual perception subtest (VMI-VP), motor co-ordination subtest (VMI-MC) | Mastery of Basic Learning Areas Questionnaire (mathematics ability rated on 4-point Likert scale) | Mastery of Basic Learning Areas Questionnaire (reading ability rated on a 4-point Likert scale) | Demographics (BMI, age, gender, SES, ethnicity) | Results for visual motor integration (VMI) are highly significant for mastery of mathematics and reading  VMI motor coordination standard score not significantly related to the mathematics score but was related to reading  Relationship with mathematics and reading stronger for VMI than for BOT-2 (short form), and to a lesser degree VMI-motor coordination | 85% |
| **Pitchford et al**  **(2016)**  **England** | Cross-sectional | n=62 year 1 children; 53% girls;  Age: 5 years, 5 months to 6 years, 8 months  Low SES areas | Bruininks Oseretsky Test of Motor Proficiency (2^nd^ Edition) – (Long Form)  (fine motor precision, fine motor integration subtests) | Wechsler Individual Achievement Test (2^nd^ Edition) (mathematical reasoning subtest) | Wechsler Individual Achievement Test (2^nd^ Edition) (word reading subtest) | SES | Significant weak-to-moderate positive associations between fine motor integration and word reading (r=0.377, p=0.003) and mathematical reasoning (r=0.569, p<0.001)  Significant moderate positive association between fine motor precision and mathematical reasoning (r=0.597, p<0.001)  Non-significant findings between fine motor precision and word reading  Fine motor integration was found to be a significant predictor of early maths ability, but not a significant predictor of early reading ability, even when cognitive abilities were taken into account | 50% |
| **Potter et al**  **(2013)**  **USA** | Cross-sectional  (sample from Early Childhood Longitudinal Study – Kindergarten Cohort) | N=19 173 kindergarten children; 49% girls; Age: M=5.62±0.36 years | Early Screening Inventory-Revised:  Fine motor skills: (replicating a gate with cube blocks; drawing a person; copying 5 simple figures) | Early Childhood Longitudinal Study – Kindergarten Cohort (mathematics assessments) | Early Childhood Longitudinal Study – Kindergarten Cohort (reading assessments) | General knowledge assessment  Demographics  (SES, parental educational expectation, gender, age, ethnicity, family structure, non-English speaking background)  Developmental factors (socio-emotional wellbeing, attentiveness, post-natal factors) | Fine motor skills were positively associated with children's reading skills, which remained significant even after controlling for other development factors  Fine motor skills had a moderately strong and positive independent association with performance on the math assessment, which remained significant after controlling for other development factors | 90% |
| **Rigoli et al**  **(2012)**  **Australia** | Cross-sectional | n=93 adolescents; 41% girls; Age: Mean=14.2±1.1 years, SD=1.1; range 12-16 years  Varying SES | Movement Assessment Battery for Children (2^nd^ Edition) (manual dexterity, aiming and catching, balance) | Wechsler Individual Achievement Test (2^nd^ Edition) Australian (numerical operations) | Wechsler Individual Achievement Test (2^nd^ Edition) Australian (word reading) | Wechsler Intelligence Scale for Children-IV (cognitive ability)  N-back task (visuospatial working memory)  Parent-rated strengths and weaknesses of ADHD symptoms and normal behavior scale | Significant weak positive associations between aiming & catching and word reading (r=0.280, p<0.01) and numerical operations (r=0.229, p<0.05)  Aiming & catching did not have a direct impact on academic achievement, it impacted via working memory  Non-significant findings for both manual dexterity and balance with word reading/numerical operations | 70% |
| **Santi et al**  **(2014)**  **USA** | Cross-sectional  (sample from a larger longitudinal study) | n=778 children in grades 1 and 2; 52% girls  Grade 1: n=617  Grade 2: n=550  SES: 8% low, 40% working, 46% middle-upper  Ethnicity: 50% white, 18% African American, 16% Hispanic, 15% Asian 1% Other | Beery-Buktenica Developmental Test of Visual-Motor Integration (3rd Edition): Visual motor integration |  | Woodcock-Johnson Psycho-Educational Battery – Revised (passage comprehension)  Formal Reading Inventory (reading comprehension)  Comprehensive Test of Phonological Processing (phonological awareness)  Woodcock-Johnson Psycho-Educational Battery – Revised: (letter word identification, word attack)    Word Reading Efficiency (fluency) | Wechsler Intelligence Scale for Children – Revised (performance / verbal IQ)  Peabody Picture Vocabulary Test – Revised (vocabulary recognition)  Woodcock-Johnson Psycho-Educational Battery – Revised: (reading vocabulary)  Rapid Automatized Naming Test (rapid naming) | Visual motor integration (VMI) skills are related to reading in both grades 1 and 2  Significant very weak-to-weak positive correlations between VMI and passage comprehension (r=0.33, p<0.0015); formal reading inventory (r=0.19, p<0.0015), phonological awareness (r=0.37, p<0.0015), letter-word identification (r=0.35, p<0.0015), word attack (r=0.33, p<0.0015) and word reading efficiency (r=0.30, p<0.0015) in Grade 1  Significant very weak-to-weak correlations between VMI and passage comprehension (r=0.21, p<0.0015); phonological awareness (r=0.32, p<0.0015), letter-word identification (r=0.30, p<0.0015), word attack (r=0.31, p<0.0015) and word reading efficiency (r=0.25, p<0.0015) in Grade 2  Non-significant findings between VMI and formal reading inventory in Grade 2 (r=0.08, p=0.059)  When other known predictors of reading are included (e.g. phonological awareness, decoding, fluency and vocabulary), contribution of VMI to reading over and above the other predictors is negligible | 65% |
| **Sortor and Kulp**  **(2003)**  **USA** | Cross-sectional | n=155 children in grades 2-4; Age: M=8.4±1.0 years; range 7 to 10 years  n=42 Grade 2  n=55 Grade 3  n=58 Grade 4  Primarily white, middle-class, suburban elementary school | Beery Visual Motor Integration Test (4^th^ Edition): Visual motor integration (VMI); VMI Supplemental Developmental Test of Motor Coordination | Stanford Achievement Test Series (9th Edition) (total math percentile) | Stanford Achievement Test Series (9th Edition) (reading percentile scores) | Otis-Lennon School Ability Test (verbal ability score / cognitive ability)  VMI Supplemental Developmental Test of Visual Perception (visual analysis/spatial skills-motor-reduced) | Significant relation between math achievement and performance on tests of VMI, visual perception and motor coordination  Partial correlations (controlling for age and verbal ability) showed there were significant associations between VMI standard score and maths (r=0.274, p=0.001) and reading (r=0.163, p=0.05)  Controlling for age and verbal ability, there were significant associations between VMI-motor coordination and maths (r=0.218, p=0.008) and reading (r=0.184, p=0.027)  Multiple regression analyses found VMI and motor coordination were not predictive of reading and maths achievement | 50% |
| **Suggate et al**  **(2017)**  **Germany** | Cross-sectional | n=81 children; 50.6% girls; Age: M=4 years 9 months, SD=11.14 months, range 39-76 months | Fine motor skills (pegboard task, bead threading, block turning) | Overall numerical skills (combination of non-finger based and finger-based numerical skills)  Non finger-based numerical skills (i.e. counting and arithmetic without using fingers)  Finger-based numerical skills (i.e. children prompted to use fingers count) |  | Parental questionnaire (children's country of birth, languages spoken at home, educational achievement at secondary and tertiary levels)  Peabody Picture Vocabulary Test-IV (German) (receptive vocabulary) | Significant strong positive associations between fine motor skills and overall numerical skills (r=0.73), finger-based numerical skills (r=0.69) and non-finger based numerical skills (r=0.70)  Linear regression analyses showed that fine motor skills (independent of age and receptive vocabulary) contributed significantly to overall numerical skills (β=0.34, p<0.05),  finger-based numerical skills (β=0.40, p<0.05), non-finger-based numerical skills (β=0.24, p<0.05)  Controlling for age, the link between FMS and non-finger-based numerical skills was mediated by finger numerical skills | 45% |
| **Suggate et al**  **(2018)**  **Germany** | Cross-sectional | n=144 children; 55.6% girls; Age: Mean=6 years, 1 month, SD=3.28 months | Movement Assessment Battery for Children (German version) (posting coins, threading beads, tracing through a maze)  Grapho-motor skills: Greek-letter copying task |  | Bielefelder Screening Test for the Identification of Early Reading difficulties (phonemic awareness)  Dynamic Indicators of Basic Early Literacy Skills (letter naming task)  Reading skill (estimated with non-word decoding, word reading and reading of high-frequency words) | Parental questionnaire (ethnicity, language spoken at home, country of birth, educational achievement)  Writing: Name writing  Wechsler Preschool and Primary Scale of Intelligence (concept recognition, general knowledge, picture concepts, expressive/ receptive vocabulary subtests)  Kaseler concentration test (attention) | Significant very weak-to-weak positive associations between phonemic awareness and coin posting (dominant hand) (r=0.19, p<0.05), coin posting (non-dominant hand) (r=0.17, p<0.05), maze tracing (r=0.18, p<0.05) and fine motor total (r=0.23, p<0.05)  Significant very weak positive associations between word reading and fine motor total (r=0.18, p<0.05)  Significant weak positive associations between grapho-motor skills and phonemic awareness (r=0.26, p<0.05), letter naming (r=0.25, p<0.05) and word reading (r=0.27, p<0.05) | 60% |
| **van der Niet et al**  **(2014)**  **Netherlands** | Cross-sectional | n=263 children; 45% girls; Age: M=9.5±1.2 years; range 7-12 years  SES: 12% low or middle low SES | European physical fitness test battery (Physical fitness)  Standing broad jump (explosive leg strength)  Sit-ups (trunk strength)  10 x 5m shuttle run (running speed and agility) | Dutch standardized test scores on maths | Dutch standardized test scores on reading | Anthropometrics (height, weight, BMI)  20m shuttle run (cardiorespiratory endurance)  Tower of London test (problem solving skills)  Trailmaking test (cognitive flexibility)  Dutch standardised test scores on spelling | Significant association between maths and time taken to complete shuttle run (r=-0.22, p<0.01)  Significant very weak-to-weak positive associations between maths and trunk strength (r=0.15, p<0.05) and leg strength (r=0.32, p<0.01)  Significant very weak positive associations between reading and leg strength (r=0.18, p<0.01)  Non-significant associations between reading and speed and agility and trunk strength | 65% |
| **Van Niekerk et al**  **(2015)**  **South Africa** | Cross-sectional  (sample from Physical Activity and Health Longitudinal study) | n=236 adolescents; 58% girls; Age: 13-14 years | Bruininks Oseretsky Test of Motor Proficiency (2^nd^ Edition) (Short Form) | End of year report (average maths marks) |  |  | Significant very weak-to-weak positive correlations for total group between maths and draw a line (r=0.16, p<0.05) and fold paper (r=0.13. p<0.05); hop on one leg (r=0.18, p<0.05); sit ups (r=0.29, p<0.05); total motor proficiency (r=0.23, p<0.05)  Non-significant findings for total group between maths and fine motor integration, manual dexterity, upper limb coordination, body coordination, balance and strength (push ups) | 35% |
| **Xiang et al**  **(2017)**  **China** | Cross-sectional | n=144 adolescents; 33% girls; Age: M=14.55±0.62 years; range 13-17 years | Skill related physical fitness:  50m dash (running speed)  Standing long jump (power) | Chinese standardized test (mathematics) | Chinese standardized test  (language literacy) | FITNESSGRAM; Health-related physical fitness (CRF, muscular fitness, body composition)  Center for Epidemiological Studies Depression Scale (Depression) | Significant weak positive association between skill-related physical fitness and mathematics (r=0.17, p<0.05) | 45% |
