# Supplementary material for: Relationships between Motor Proficiency and Academic Performance in Mathematics and Reading in School-Aged Children and Adolescents: A Systematic Review
Source: Int J Environ Res Public Health. 2018 Jul 28;15(8):1603. doi: 10.3390/ijerph15081603 (PMC6121293; doi:10.3390/ijerph15081603)
Supplement: Supplementary file 1 [file ijerph-15-01603-s001.zip › MACDONALD_MILNE_ORR_POPE_Supplementary Table S2_FINAL.docx]

Table S2: Key data extracted from experimental studies examining the impact of motor proficiency-related interventions on academic performance in mathematics and reading in school-aged children and adolescents

| Authors (Year), Country | Study Design | Study Participants  Sample size;  (% girls); Age (Mean ± SD; range); schools (n); classes (n); SES;  Ethnicity | Outcome Measures | | | Covariates | Intervention  (dose, frequency, duration, groups) | Main Findings | Critical Appraisal Percentage  (%) |
| --- | --- | --- | --- | --- | --- | --- | --- | --- | --- |
|  |  |  | Motor proficiency | Academic performance (mathematics) | Academic performance (reading) |  |  |  |  |
| Beck et al  (2016)  Denmark | Experimental  Cluster randomized intervention  6-week intervention with 8-week follow up | Total: n=165; 47% girls; Age: M=7.5±0.02 years  n=3 Danish public schools; n=9 year 1 school classes  Control group (CON): n=57 participants; 50.9% girls; Age: M=7.5 ± 0.02 years  Fine motor math group (FMM): n=53; 43.4% girls; Age: M=7.5±0.03 years  Gross motor math group (GMM): n=55; 45.5% girls; Age: M=7.5±0.02 years | Coordination Wall (gross motor skills; movements performed require crossing of the vertical midline)  Perdue Pegboard Test (fine motor skills; manual dexterity, bimanual fine motor coordination)  Assessments occurred before (T0), immediately after (T1) and 8 weeks after the intervention (T2) | Mathematical test (standardized, diagnostic test in Denmark)  Assessments occurred before (T0), immediately after (T1) and 8 weeks after the intervention (T2) |  | Cognitive Tests  Modified Eriksen Flanker Test (executive function)  Cambridge Neuro-psychological Test Automated Battery - Spatial span test (visuo-spatial short term memory)  Free-recall wordlist memory test (phonological short term memory)  Andersen Test (CV fitness)  Physical load during intervention (heart rate (HR) monitoring; accelerometers - time spent in low, moderate-vigorous HR zones) | 60-minute mathematics lessons, 3 x week, 6 continuous weeks. Delivered by classroom teacher.  CON: Received non-motor enriched conventional mathematical teaching  FMM group: Mathematical teaching enriched with fine motor activity  GMM group: Mathematical teaching enriched with gross motor activity  To standardize within and between intervention groups, research staff delivered three workshops of three hours to classroom teachers. Teachers received teaching manuals. Regular communication between research staff and classroom teachers | Applying gross motor enriched math lessons resulted in a greater improvement in mathematical performance compared to fine motor enriched math and conventional math lessons  All groups improved their mathematical performance T0 to T1. Changes in mean mathematical performance significantly greater in GMM compared to FMM (1.87 ± 0.71 correct answers, p=0.02). No significant differences in mathematical performance observed at T2  Subgroup analyses revealed normal math performers benefitted from GMM compared to both CON and FMM (not observed in low math performers)  Changes in gross motor performance accounted for ~ 25% of the effects of the intervention on mathematical performance  Changes in fine motor performance accounted for ~ 10.7% of the effects of the intervention on mathematical performance | 71% |
| Callcott et al  (2015)  Australia | Quasi -experimental – controlled pre-post design  Intervention delivered over one school year | Initially n=400 (100 per group); Age: between 4-5 years  n=8 primary schools  Middle to high Index of Community Socio-educational Advantage  Literacy + Movement Group (Lit +Movt): n=85  Literacy Group (Lit only): n=67  Movement Group (Movt only): n=67  Control (Con): n=79 | Movement Assessment Battery for Children-2^nd^ Edition (MABC-2) (manual dexterity, aiming and catching, balance) |  | Test of Phonological Awareness (PA) | Developmental Spelling Test  Wide range Achievement Test - Revised: (spelling subtest) | Intervention delivered by classroom teacher  Lit + Movt Group: Let's Decode (phonological awareness and systematic decoding instruction - 15 min each day) + Moving on with Literacy (30 action songs with movement challenge and language - 15 min per day), 5 days per week, school terms 1 to 4  Lit Group: Let's Decode – 15 min per day, 5 days per week, school terms 1 to 4  Movt Group: Moving on with Literacy – 15 min per day, 5 days per week, school terms 1 to 4  Control Group: classroom teachers conducted their regular pre-primary program, which included PE and English  Research staff delivered professional development sessions to classroom teachers to teach Let’s Decode and Moving on with Literacy programs. Research staff visited each classroom teacher and observed them teaching programs and visited once per term. | Lit + Movt group's average post-test performance on phonological awareness test was better than each of the other groups (Lit only, Movt only, Con)  Significant main effect of the intervention after controlling for pre-test differences (p=0.001). Lit + Movt group performed significantly better than their peers in both the Movt only (p=0.003) and Con (p=0.001)  Students in the Lit + Movt group performed better at post-test on the MABC-2 test and made the largest average gains than the other groups. Significant difference found between the Lit + Movt group and Con group. | 50% |
| Erasmus et al  (2016)  South Africa | Quasi-experimental – controlled pre-post design  10-week intervention | n=48; Age: 5-6 years  n=2 schools, n=2 classes  Experimental group (Exp): n=21; 52% girls; Quintile 1 school (low SES)  Control group (Con): n=27; 59% girls; Quintile 2 school (low SES) | La Roux's Group Test for School Readiness:  Fine motor ability (completion of a maze and writing patterns);  Gross motor coordination: (Single leg stance (SLS)-eyes open/closed) hop, walking heel-to-toe on straight line)  (Pre / post- test) | La Roux's Group Test for School Readiness:  Number concept subtest (counting concrete objects, amounts and relationships)  (Pre / post- test) |  | La Roux's Group Test for School Readiness (5 subtests)  Visual perception; spatial orientation; language experience; drawing human figure; auditory perception | Intervention program delivered by research staff for 40 minutes each lesson, 3 x a week for 10 weeks  Intervention program included gross motor (20 min), fine motor (10 min) and perceptual exercises (visual and auditory discrimination, spatial orientation, midline crossing – 10 min) | Experimental group improved significantly in the sub-items for number concept (p<0.012, Cohen’s d effect size d=1.13), gross motor coordination (p<0.01, Cohen’s d effect size d=2.16). Improvements in the number concept subtest were not significantly better than the control group following the intervention (when controlling for differences in pre-test scores)  Experimental group scored significantly better in the post-test (moderate and large effects) than the control group in visual perception, language experiences, gross motor coordination (p=0.0088, Cohen’s d effect size d=2.08) and total score (p=0.0089, Cohen’s d effect size d=0.80) | 54% |
| Ericsson  (2008)  Sweden | Quasi-experimental, non-randomized,  controlled design  3-year intervention | n=251; age: 7-9 years  School: n=1  Two intervention groups (n=152); one control group (n=99) | MUGI Observation Checklist  (16 gross motor tasks: skip jumping, hopping, SLS, throwing, dribbling, catching; measuring 2 variables of motor skills (balance/ bilateral coordination) | Lus Test (national test in mathematics) | Lus test (reading development, word test  reading test) | Conner's questionnaire (attention and impulse control)  Questionnaire to parents (father/mother education, income, attitudes to PA, amount of PA in spare time (children and parents) | Intervention group:  60-minute lesson of Physical Education (PE), 5 lessons per week ± one extra 45-minute lesson of motor training per week. Lessons under the supervision of the school's PE teacher (3 lessons per week) or local sports clubs (2 lessons per week)  Control group:  Usual 60-minute PE lesson, 2 lessons per week | After 2 years, pupils in intervention group had better results than pupils in control group in national tests for Swedish with overall large differences in results between groups (Cramer’s index 0.29)  Pupils in intervention group had better results in national mathematics tests than control with overall small differences in results between groups (Cramer’s index 0.21)  There were significant differences in academic performance between pupils with good motor skills and pupils with deficits in motor skills in both the intervention and control groups  Pupils in intervention group had better motor skills than pupils in the control group (after 1 year difference between groups was large - Cramer's index 0.24; after three years, differences were very large Cramer’s index 0.37) | 25% |
